# Epidemiological and geospatial profile of the prescription opioid crisis in Ohio, United States

**DOI:** 10.1038/s41598-020-61281-y

**Published:** 2020-03-09

**Authors:** Andres Hernandez, Adam J. Branscum, Jingjing Li, Neil J. MacKinnon, Ana L. Hincapie, Diego F. Cuadros

**Affiliations:** 1 Department of Geography and Geographic Information Science, University of Cincinnati, Cincinnati, USA; 2 Health Geography and Disease Modeling Laboratory, University of Cincinnati, Cincinnati, USA; 3 Department of Biostatistics, College of Public Health and Human Sciences, Oregon State University, Corvallis, USA; 4 Urban Health Collaborative, Dornsife School of Public Health, Drexel University, Philadelphia, Pennsylvania USA; 5 James L. Winkle College of Pharmacy, University of Cincinnati, Cincinnati, Ohio USA

**Keywords:** Public health, Epidemiology, Risk factors

## Abstract

The underlying reasons behind the unprecedented increase of the mortality rates due to the opioid epidemics in the United States are still not fully uncovered. Most efforts have been focused on targeting opioids, but there is little information about vulnerable populations at high risk of opioid abuse and death. In this study, we used data from the Ohio Department of Health for deaths caused by prescription opioids from 2010–2017 to analyze the spatiotemporal dynamics of the opioid overdose epidemic. Our results showed a rapid increase in prescription opioid death rates among the white male population aged 30–39 but also a considerable increase among the black male population with an exponential growth trend. Our geospatial analysis suggests that the increasing rates of the opioid overdose epidemic in Ohio were driven by the epidemic hotspot areas. Our findings highlight the relevance of prioritizing public health measures targeting specific locations and vulnerable populations to mitigate the current opioids crisis.

## Introduction

The United States (US) is currently experiencing an opioid overdose crisis with an unprecedented magnitude. Adjusted for age, the opioid-related death rate of 21.7 deaths per 100,000 people in 2017, and 20.7 in 2018 were the highest worldwide^1^. According to the Centers for Disease Control and Prevention (CDC), 67,367 deaths by drug overdose occurred in the US during 2018, and 351,564 deaths were related to opioids resulting in 0.36 years of life expectancy lost in 2016^1,2^. The major cause of death among people under 50 years old in the US in 2017 was drug overdose, exceeding the rates of death caused by motor vehicle and firearms^3^. Opioids have become a widespread cause of accidental fatal overdose, which historically were attributed to heroin and prescription opioid pain relievers. Recent reports show that overdoses caused by synthetic opioids (e.g., fentanyl and analogues) are emerging as a national public health emergency, as declared by the US Department of Health and Human Services in 2018^4^.

National data on opioid overdose mortality rates show that the epidemic is not homogenously distributed within the US. Twenty states and the District of Columbia have reported age-adjusted drug mortality rates that are statistically higher than the national rate. Among these, West Virginia (51.5 deaths per 100,000 inhabitants), Delaware (43.8), Maryland (37.2), Pennsylvania (36.1.3), Ohio (35.9), and New Hampshire (35.8) had the highest age-adjusted drug overdose rates in 2018^1^. Moreover, Ohio is one of eight states with a doubling of the opioid mortality rate every three years from 1999 to 2016, and recently has experienced an unprecedented number of deaths caused by unintentional drug overdose, especially deaths caused by synthetic opioids^2,5^. Specifically, there was a 169% increase from 1,544 deaths in 2010 to 4,157 deaths in 2017, and approximately 13,000 overdose events reversed by the use of naloxone.

Several reasons are attributed to the geographical disparity of the opioid overdose mortality^6–8^. Among the potential factors of the uneven distribution, special attention has been devoted to the association between the opioid overdose mortality and drug prescription rates, and the availability of medications to reverse overdose events (naloxone)^8^. This approach can be linked to the intervention measures implemented in these high opioid overdose burden areas to tackle the epidemic, which are focused on controlling prescription while increasing the availability of resources to treat the adverse effects of the overdose events^9^. Additionally, other studies also propose socioeconomic characteristics associated with the high-risk opioid overdose areas^10^. Health accessibility (primary care and mental health accessibility), unemployment rate, urbanicity, and availability of prescription vs. non-prescription opioids seem to be associated with high rates of opioid overdose mortality^7,8^. However, the reasons behind the uneven spatial distribution of the opioid epidemic in the US are still not well understood. Although the link between the number of opioid prescriptions and opioid overdose deaths has been observed, explanations about the rapid rise in the epidemic in specific areas remain incomplete, especially in areas where the number of opioid prescriptions is low and the determinants (i.e., drivers) of the epidemic have not been completely established^11^. Moreover, the epidemiological characteristics of the opioid public health crisis remain understudied and there is a lack of substantial spatial analysis for allowing health authorities to make decisions for resource allocation at small scales.

While most efforts have been focused on targeting opioids, there is little information about vulnerable populations at high risk of opioid abuse and death, where and what demographical groups are at higher risk, and what are the drivers boosting the epidemic of opioid overdose deaths in the recent years^12^.

In this context, geospatial statistical and epidemiological models are important tools for identifying the spatial and temporal dynamics of the epidemic^13^. Understanding the critical spatiotemporal characteristics of the opioid overdose crisis will provide valuable information to identify the potential socio-economic drivers of the epidemic as well as geographic areas where vulnerable populations are located, and where interventions should be implemented.

In this study, we used data from the Ohio Department of Health for deaths caused by prescription opioids from 2010–2017 to analyze the spatiotemporal dynamics of the opioid overdose epidemic in Ohio. The objective of the present paper is threefold, namely, to identify (i) the demographic groups in Ohio at higher risk of prescription opioid overdose death, (ii) the geographic areas in Ohio where the burden of overdose mortality is concentrated, and (iii) the temporal trend of the opioid epidemic in Ohio. The results from this study will inform public health authorities and clinical practitioners about which locations and groups should be prioritized for surveillance to improve public health response addressed to mitigate the current opioids crisis, not only in Ohio but also inform other parts of the country experiencing emerging epidemics, and even other countries of the world where opioid epidemics seems to be emerging.

## Results

### General profile of the opioid overdose epidemic

**Figure 1:**
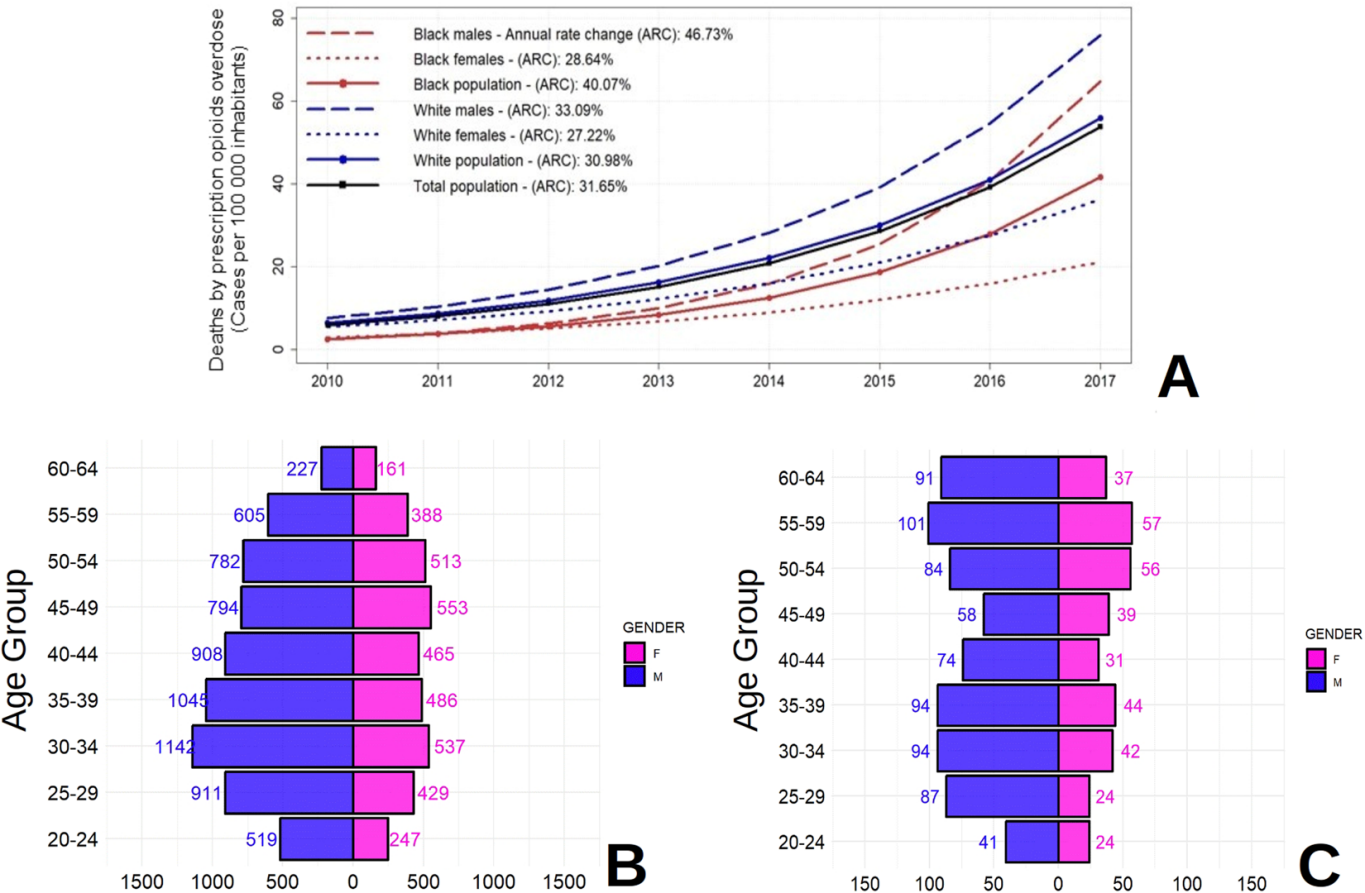
(**A**) Descriptive demographics and annual rate change (ARC) of prescription opioids overdose mortality rates by major demographic groups in Ohio (2010–2017). (**B**) Case counts by age groups for white population. (**C**) Case counts by age groups for black population.

Deaths by unintentional prescription opioids overdose in Ohio for 2010–2017 resulted in 11,790 cases (white population: 10,712 – black population: 1,078). Deaths have increased continuously from 653 in 2010 to 3,674 in 2017, with an exponential growth trend. Prescription opioid overdose mortality rates increased from 9.98 to 57.31 per 100,000 inhabitants from 2010 to 2017 (Table 1). The highest prescription opioid overdose mortality rates were found in the white male population aged 30–34, with 131.45 cases per 100,000 inhabitants, followed by males aged 35–39 with 120.48 cases per 100,000 inhabitants, compared to the 57.31 cases per 100,000 inhabitants for the total population in 2017 (Table 1). Figure 1 illustrates the temporal growth trends for race and gender categories and shows that black males were experiencing the fastest estimated annual increase (46.73%) of the opioid overdose mortality rate, compared to the total population rate (31.65%) during the period of study. For this specific race group, the highest rate was found in black males aged 35–39, with 109.55 cases per 100,000 inhabitants in 2017.

**Table 1:** Descriptive demography of proportion (%) for deaths by prescription opioid overdose deaths in Ohio (2010–2017).

RACE	GENDER	AGE GROUP	2010	2011	2012	2013	2014	2015	2016	2017
Black	Female	20–24	1.67	0.00	1.52	0.00	1.46	4.45	7.62	20.36
Black	Female	25–29	0.00	1.90	3.76	5.49	1.74	3.29	12.48	10.37
Black	Female	30–34	6.07	3.97	0.00	1.92	5.76	7.67	17.00	37.05
Black	Female	35–39	2.05	6.37	2.17	0.00	12.78	8.27	18.16	39.21
Black	Female	40–44	8.37	4.12	2.03	2.02	4.08	10.45	15.14	19.78
Black	Female	45–49	1.92	1.98	6.17	6.36	10.81	12.94	19.00	22.87
Black	Female	50–54	14.83	5.56	14.98	13.33	9.69	9.94	12.31	29.62
Black	Female	55–59	8.85	10.75	8.34	6.06	7.93	17.58	25.36	29.47
Black	Female	60–64	5.63	5.26	7.70	5.04	12.22	7.12	27.62	17.78
**Black**	**Female**	**All**	**5.39**	**4.22**	**5.06**	**4.36**	**6.91**	**8.79**	**16.62**	**24.73**
Black	Male	20–24	0.00	3.32	4.68	1.48	4.36	10.19	14.74	22.55
Black	Male	25–29	2.14	4.24	4.16	7.95	7.48	19.33	47.73	52.61
Black	Male	30–34	4.53	4.46	6.61	4.33	17.35	34.48	57.13	70.00
Black	Male	35–39	6.83	2.36	4.82	0.00	16.48	29.80	40.25	109.55
Black	Male	40–44	2.30	4.53	2.25	4.49	13.62	18.68	31.28	100.25
Black	Male	45–49	4.32	4.45	9.14	11.69	2.39	18.86	34.79	48.04
Black	Male	50–54	8.41	14.67	10.57	8.58	17.45	11.17	25.13	93.78
Black	Male	55–59	5.12	17.45	9.64	18.71	22.90	31.54	51.59	74.59
Black	Male	60–64	0.00	0.00	12.59	21.44	17.77	39.91	55.29	106.70
**Black**	**Male**	**All**	**3.78**	**6.21**	**6.87**	**7.96**	**12.62**	**22.58**	**38.62**	**70.91**
**Black**	**All**	**All**	**4.63**	**5.16**	**5.92**	**6.07**	**9.63**	**15.37**	**27.14**	**46.83**
White	Female	20–24	4.31	2.62	2.60	2.60	9.18	14.36	21.78	25.91
White	Female	25–29	6.65	7.38	7.79	7.76	13.19	23.30	36.12	43.88
White	Female	30–34	7.21	8.17	12.35	11.57	12.99	22.20	44.24	71.31
White	Female	35–39	9.33	8.32	8.14	9.27	16.59	27.33	44.46	51.41
White	Female	40–44	11.02	10.72	10.25	10.14	16.88	25.91	27.11	50.52
White	Female	45–49	13.20	15.98	16.55	12.25	15.79	23.43	32.14	42.38
White	Female	50–54	8.59	11.72	10.06	14.32	15.42	23.18	27.91	33.73
White	Female	55–59	6.01	6.48	8.01	10.06	9.45	15.14	21.93	29.15
White	Female	60–64	2.63	1.87	2.52	2.81	11.08	6.33	10.09	11.34
**White**	**Female**	**All**	**7.79**	**8.32**	**8.77**	**9.10**	**13.30**	**19.81**	**28.90**	**38.84**
White	Male	20–24	10.12	9.02	6.69	7.63	15.07	24.71	47.15	49.84
White	Male	25–29	13.61	15.04	11.89	14.47	26.29	45.18	80.33	95.13
White	Male	30–34	21.61	16.67	17.61	17.16	35.44	64.09	97.89	131.45
White	Male	35–39	16.01	14.58	20.10	14.16	38.42	54.72	97.81	120.48
White	Male	40–44	16.47	11.42	17.73	17.49	26.92	49.14	77.72	107.20
White	Male	45–49	16.31	18.94	14.14	15.82	19.47	34.63	54.79	77.64
White	Male	50–54	15.27	16.66	12.84	17.57	22.41	26.19	47.70	70.33
White	Male	55–59	9.22	8.18	6.89	8.48	18.83	24.80	42.14	54.04
White	Male	60–64	5.57	1.66	4.01	3.97	6.84	8.62	16.00	25.57
**White**	**Male**	**All**	**13.80**	**12.52**	**12.25**	**12.94**	**22.86**	**35.91**	**60.87**	**79.43**
**White**	**All**	**All**	**10.77**	**10.40**	**10.49**	**11.00**	**18.05**	**27.81**	**44.80**	**59.04**
**All**	**All**	**All**	**9.98**	**9.72**	**9.89**	**10.34**	**16.90**	**26.10**	**42.33**	**57.31**

### Spatial clustering analysis of the opioid overdose epidemic

**Figure 2:**
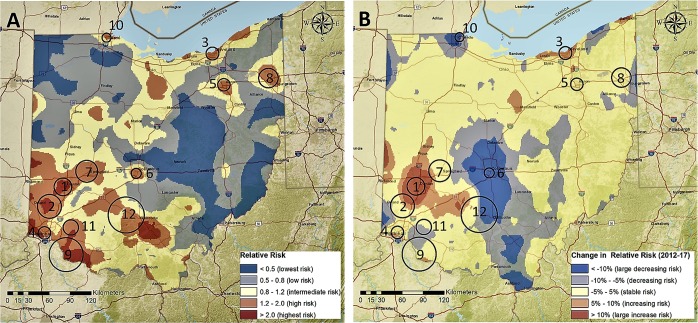
(**A**) Spatial distribution of relative risk for death by prescription opioids overdose in Ohio (2010–2017) with identified clusters of opioids overdoses. (**B**) Change of the relative risk (First semester 2010 compared to Last semester 2017) with identified clusters of opioids overdoses. Maps were created using ArcGIS by Esri version 10.5 (http://www.esri.com)^38^, and basemaps were obtained from ESRI and National Geographic available at ArcGIS Online basemaps^39^.

We identified 12 geographical clusters (hotspots) of prescription opioid overdose mortality cases where most of the burden of the epidemic in Ohio was concentrated during 2010–2017 (Table 2). These 12 hotspots encompassed 21% of the population at risk (1,363,811) and contained 4,769 (40%) total deaths by prescription opioids overdose from 2010 to 2017. In 2017, mortality rates by prescription opioid overdose were almost three times higher within the hotspots compared with the outside areas, with 116.00 cases per 100,000 inhabitants within the hotspot areas and 40.49 cases per 100,000 inhabitants outside of the hotspots. The geographical locations of the identified hotspots are presented in Fig. 2. Most hotspots (Clusters 1, 2, 4, 6, 7, 9, 11, and 12) were located around the three major southwestern cities of Ohio (Dayton, Cincinnati, and Columbus). Three additional hotspots (Clusters 3, 5, and 8) were identified in the cities of Cleveland, Akron, and Youngstown, and one small hotspot (Cluster 10) was identified in the city of Toledo on the northern border with Michigan.

**Table 2:** Identified clusters of deaths by prescription opioid overdose for Ohio 2010–2017, and aggregations by hotspots (HS) and Non-Hotspots (NHS) areas. Confidence Intervals (CI) at 95% are included for averaged relative risks (RR) and RR temporal change of mortality rate of prescription opioid overdose.

Cluster	Radius (Km)	Estimated population in 2017	Total death cases (All years)	Total death cases (2017)	Mortality rate in 2017 Cases per 100.000 hab.	RR (2017) Mean [95% CI]	RR temporal change^a^ (%) Mean [95% CI]
1	12.64	188,097	771	353	187.66	3.61 [2.33–4.89]	+48.94 [+25.72–+72.16]
2	17.09	152,828	647	211	138.06	2.20 [1.76–2.64]	+9.63 [−10.69–+30.22]
3	9.20	165,470	607	216	130.54	2.94 [1.83–4.05]	+20.58 [−0.98–+42.16]
4	8.92	181,154	598	194	107.09	2.61 [1.90–3.31]	+7.87 [−2.55–+18.30]
5	8.87	127,772	437	105	82.18	2.58 [1.90–3.26]	+17.88 [+4.55–+31.20]
6	7.14	112,734	386	121	107.33	2.45 [0.63–4.26]	–16.99 [−33.85––0.12]
7	15.66	69,965	254	82	117.20	1.78 [1.20–2.36]	+1.77 [−9.77–+13.31]
8	13.93	125,693	381	128	101.84	1.78 [1.37–2.18]	+1.18 [−7.76–+10.12]
9	24.34	49,170	173	46	93.55	2.41 [1.51–3.31]	−2.10 [−12.66–+8.47]
10	6.44	120,373	315	78	64.80	1.71 [1.26–2.17]	−9.07 [–19.49–+1.36]
11	10.96	8,672	37	7	80.72	3.04 [0.77–5.28]	−1.65 [−15.58–+12.28]
12	25.83	61,884	163	41	66.25	1.28 [0.76–1.81]	−2.84 [−25.20–19.53]
**TOTAL HS**	161.01	1,363,811	4,769	1,582	116.00	2.42 [2.15–2.68]	+10.17 [+4.82–+15.51]
**TOTAL NHS**		5,166,333	7,021	2,092	40.49	0.80 [0.77–0.84]	−3.20 [−4.10–−2.31]
**TOTAL**		6,530,144	11,790	3,674	56.26	1.01 [0.95–1.06]	−1.51 [–2.57–−0.45]

^a^RR temporal change 2010–2017 was defined as: $\frac{(\overline{{RR}}{}_{{last}{semester}2017}\,-\,{\overline{{RR}}}_{{first}{semester}2010})}{{\overline{{RR}}}_{{first}{semester}2010}}\ast$.

### Space and space-time relative risk estimation

The spatial distribution of the relative risk (RR) of death by prescription opioid overdose is presented in Fig. 2A. RR ranged from 0 to 8.16, and was classified as lowest risk areas (RR < 0.50), low risk (RR: 0.50–0.80), intermediate risk (RR: 0.80–1.20), high risk (RR: 1.20–2.00), and highest risk (RR > 2.00). In 2017, the RR within the hotspot areas was significantly higher (average RR = 2.42, 95% confidence interval [CI] 2.15–2.68) compared to the non-hotspot areas (RR = 0.80, 95% CI: 0.77–0.84). Additionally, the RR was significantly higher than 1 in most of the hotspots except for Cluster 6 (RR = 2.45, 95% CI: 0.63–4.26), Cluster 11 (RR = 2.20, 95% CI: 0.77–5.28), and Cluster 12 (RR = 1.28, 95% CI: 0.76–1.81). The highest RR values were in Cluster 1 (RR = 3.61, 95% CI: 2.33–4.89) around the city of Dayton, followed by Cluster 3 (RR = 2.94, 95% CI: 1.83–4.05) in Cleveland, and Cluster 4 located in the Cincinnati area (RR 2.61, 95% CI: 1.90–3.31).

Temporal analysis of the RR of death caused by prescription opioid overdose identified a significant increase over time within the hotspot areas (RR change from 2010 to 2017: +10.17%, 95% CI: +4.82–+15.51%) (Fig. 2B). Conversely, RR in the non-hotspots areas was significantly decreasing (RR Change: −3.20%, 95% CI: −4.10–−2.31%). The highest RR increase was observed in Cluster 1, with an RR increase of 48.94% (95% CI: 25.72–72.16%). The only hotspot for which RR had a significant reduction was Cluster 6 located within the area of Columbus, Ohio (RR Change: −16.99%, 95% CI: −33.85–−0.12%).

### Temporal trend analysis

**Figure 3:**
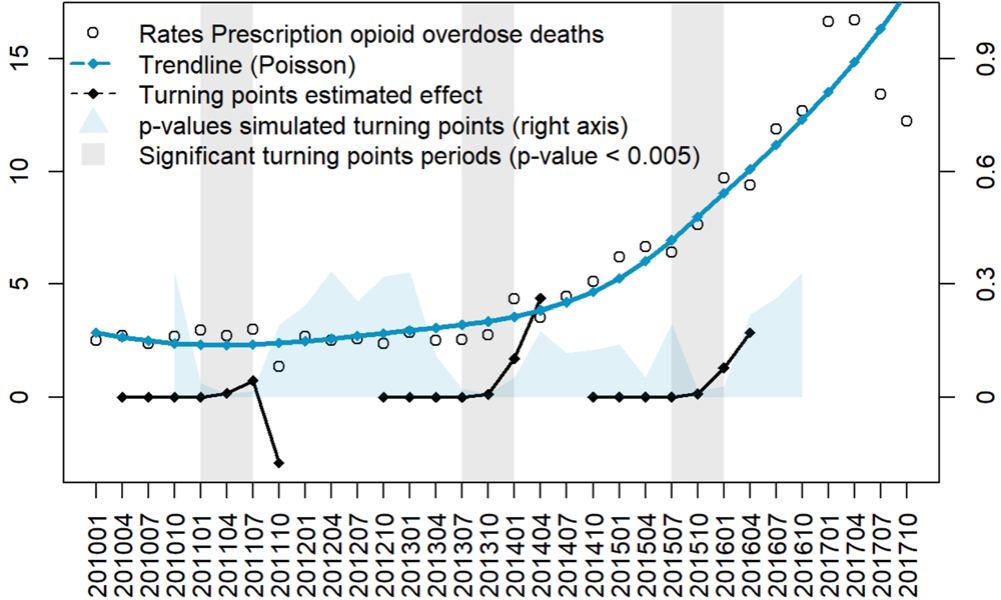
Ohio prescription opioid overdose death rates (cases per 100,000 inhabitants) aggregated by trimester (from January 2010 to October 2017). Percentual causal effect estimation for trimesters with significant changes (p-value < 0.005) over a period of 24 months are included in the black trends. P-values for the simulated turning points from October 2010 to October 2016 are described in the blue area (scaled to the right axis).

Temporal trend analysis identified three significant changing trends of the opioids overdose epidemic in Ohio. Figure 3 shows the cumulative percentage difference between the observed growth rate of death rates by prescription opioid overdose and the expected counterfactual in the significant trimesters (dashed, black lines). The first trend was identified in January 2011 with a decreasing effect in the trajectory of the epidemic (p < 0.005). Conversely, two trends with an increasing effect in the mortality rate were identified in July 2013 and October 2015 (p < 0.005). The increasing trend identified in July 2013 had the highest impact in the temporal dynamics of the epidemic, with an estimated difference between the observed and expected changes in the mortality rate of +21.00% (95% CI: +6.10–+37.00%). This result indicates that in absence of any disturbance, we would have an expected decrease of the mortality rate of −3.00% (95% CI: −19.00–+12.00%) for each month, but in contrast we observed a monthly average increase of +18.00% starting in this time point (July 2013). Similarly, effects for the second trending increase in 2015 corresponded to a difference between observed and expected mortality rate change of +13.00% (95% CI: −3.00–+30.00%). Detailed results of temporal trending causal effect estimation can be found in Supplementary Table 1, and spatio-temporal dynamics of the opioid overdose cases are illustrated in Supplementary Video 1 in Supplementary Materials.

## Discussion

We combined a novel spatial epidemiology approach with data for prescription opioid overdose deaths from 2010–2017 from Ohio and determined that the opioid overdose epidemic is concentrated in specific locations and severely affecting specific demographic groups. We found that the burden of deaths related to prescription opioid abuse was concentrated mostly in the white male population aged 30–39, followed by black males aged 35–44, especially in Southwestern Ohio, with a remarkable exponential increasing trend. Sociodemographic risk factors have been related to substance abuse disorders by several studies conducted previously^14^. These studies found that age, gender, race, and economic factors have a significant impact in the risk of substance abuse disorders^15^. Moreover, the opioid use disorder (OUD) epidemic currently experienced in Ohio can be the result of a long complex series of epidemics related to substance abuse that have been reported in the U.S. since the early 20th century. These epidemics have several interconnected elements at individual and interpersonal level, as well as with the relationship between individuals, families and their communities^14^.

Opioid overdose deaths are often the result of a previous history of substance abuse, which often starts between the ages of 18 to 25 years^16^. Adolescents with a family history of substance abuse are 10-fold more likely to suffer drug abuse disorders in the future than other individuals^14^. Individuals aged 25–44 years may start using opioids for medical or recreational purposes, and then shift to more cost-effective substances like fentanyl^17^. Such early onset of substance addiction is consistent with our results, which show that the young adult population (25–39 years) was experiencing the highest burden of opioid overdose deaths, possibly in the last stage of an OUD history. Also, consumption of synthetic opioids such as fentanyl and analogs, which are the current leading substances associated with overdose deaths, is more frequent in white non-Hispanic males.

Our results suggest notable disparity among gender and racial groups. This could be due to the opioid overdose epidemic following a temporal trend that is comprised of three waves^14,16^. Briefly, the first wave of the opioid overdose epidemic, from 1970 to 1999, was driven by heroin overdoses, which showed a larger burden centered in the black population. The second wave, from 1999 to 2010, was mainly caused by prescription opioids often used for pain management therapy, which produced a steeper increase in death rates among the white population. This increase was driven in part by broader access to healthcare, marketing campaigns, and socio-economic determinants, as well as the marketing of opioid products to medical doctors, which was found to be associated with increased opioid prescribing and, subsequently, with elevated mortality from overdoses^18,19^. Finally, the third wave, which includes the current study time period (2010 to 2017), is mainly attributed to the combination of prescription opioids and the introduction of synthetic opioids^16,18^. Nationwide, this last wave has shown a similar increase in both racial groups (white and black)^18^.

Our results showed a rapid increase in prescription opioid death rates among the white male population but also a larger increase among the black male population. However, the underlying causes of these epidemiological trends differ among these demographic groups^14,18^. Misuse of synthetic opioids differs by gender and race. Moreover, previous analysis of healthcare accessibility among major racial groups suggests that black males are less likely to get appropriate treatment and effective medication for OUD. Black males were also reported to have a higher rate of overdose deaths caused by cocaine compared to other demographic groups^14,18^. However, this trend is rapidly changing, due in part by the current state of the opioid epidemic in which the price of synthetic opioids is decreasing while demand is increasing^16^. The access to addiction treatment and medications to reverse the effects of opioids addiction (naloxone) could also contribute to the racial disparity in the temporal trends of the opioid epidemic^20^.

We found a rapid and large increase in the rate of prescription opioids overdose death in the male population. Although, gender disparities for the OUD epidemics have shown that females are more likely to get prescription opioids than men^14^, the high amount of overdose deaths caused by synthetic drug overdoses since 2013 tends to obscure death rates caused by other types of opioids. The countermeasures to limit the effects of the opioid epidemic focusing on limiting prescriptions have resulted in declining overdose deaths by prescription opioids mainly in the female population^16^. Finally, females were found to be more likely to obtain a naloxone prescription than males^21^.

Our geospatial analysis suggests that the increasing rates of the opioid overdose epidemic in Ohio were driven by the epidemic hotspot areas. The results highlight the need for identifying areas with high and low risks when analyzing the overall epidemiological trends of the opioid crisis^22^. In fact, intrinsic spatial dynamics of epidemics identified in previous studies suggest that an overall decreasing trend in the epidemic at large geographical scales could mask local disparities with sustained or increasing burden of the epidemic in hotspot areas^22,23^. For example, whereas the RR slightly declined in the areas outside of the clusters identified, the highest RR was found in Cluster 1 (RR = 3.61), which was also the cluster with the highest temporal increase between 2010 and 2017 (RR increase: 48.94%). This area includes the city of Dayton, the city with the highest death rate due to drug overdose in the U.S. for 2017^24,25^. Moreover, reasons for the spatial concentration of opioid overdoses in the southwestern counties could be partially explained by the high amount of opioids seizures recorded in Ohio, Kentucky and Indiana, which suggest that state borders may be areas where opioid consumption for non-medical purposes (especially illegally manufactured fentanyl) is common^26^. Geographic differences in the opioid demand are also linked to the historic substance abuse burden, exacerbated by areas where job and educational opportunities have been traditionally more difficult to access^10^.

The opioid overdose epidemic may continue to increase over time. Therefore, identifying social determinants is critical to mitigate the current growing phase of the epidemic^27^. Our temporal analysis identified two periods with significant increasing trends in death rates by prescription opioid overdose. Among these periods, the steepest increase in death rates was found in July 2013. An additional discrimination analysis by drug involved in the overdose showed that death rates due to opioids excluding fentanyl had a decreasing trend until mid-2013^28^. From this time point, fentanyl-related overdoses started increasing mortality rates until 2017. Moreover, we identified an increasing trend in mortality rate over time, growing from 10.34 cases per 100,000 inhabitants in 2013 to 16.90 in 2014. The second significant increase trend in opioid related mortality rate was found during July 2015, potentially associated with the introduction of carfentanyl, which is an ultra-potent fentanyl analog known to be approved only for veterinary use^29,30^. The highest increase in mortality rates took place during this period, from 26.10 cases per 100,000 inhabitants in 2015 to 42.33 in 2016. These temporal dynamics of the opioid overdose epidemic are consistent with the time frame when the federal government classified opioid painkillers from Schedule 3 to Schedule 2 in 2014, limiting its access to both prescribers and patients. The abuse of opioids painkillers plateaued after this period, but this change also might have boosted heroin and fentanyl overdoses that increased significantly due to a rebound effect^31^. These results suggest that the opioid crisis is a complex epidemic, potentially caused by excessive prescription of pain reliever medications, but also driven by the recent inclusion of synthetic opioids for recreational use, especially involving fentanyl and analogs.

This research study has some limitations. Data from the Ohio Department of Health do not distinguish between prescription opioids for medical use and abuse by illicit manufactured synthetic opioids. Several reports suggest that many drug abusers are not aware that cocaine and heroin are being contaminated with fentanyl^16^, and deaths by overdose after consuming multiple drugs at the same time may be classified as opioid overdose. Additionally, we limited our causal trend analysis to data from January 2011 to December 2016. Some reports have indicated a decrease in deaths by opioid overdose in Ohio during the last semester of 2017 and early 2018, thought to be caused by decreased availability of carfentanyl in the illegal market and the wider availability of naloxone in Ohio and nationwide^21,24^. However, data for opioid overdose mortality data for 2018 are still not available, and there are no official reports for deaths in Ohio by drug overdose for 2018 currently available.

Despite these limitations, our study explored the epidemiological and spatiotemporal dynamics of the opioid overdose epidemic in Ohio from 2010 to 2017. We identified specific geographic areas in Ohio where the epidemic was concentrated during this time period. The methodology used and the findings derived from them are of fundamental importance, particularly for identifying and preventing the emergence of similar drug abuse epidemics in the country and worldwide^10^. For example, oxycodone, known to be one of the first prescription opioid used for pain management, is suspected to be the main driver of emerging substance addiction epidemics not only in Canada, UK, and Australia, but also in China, Brazil, Colombia, Egypt, Mexico, Philippines, Singapore, South Korea, South Africa, and Spain^10^.

Methods to eradicate the opioid epidemic include targeting the socio-economic and demographic drivers of the epidemic, together with projects to increase access to naloxone and the creation of safe places for controlled drug use to help avoid death and prevent the spread of other diseases^10,27^. Additionally, tighter controls on prescriptions and the flow of illicitly manufactured fentanyl to the black market are starting to force a declining trend in overdose death rates^9,16^. Strategies to monitor the amount of fentanyl diluted in other drugs and disseminating information among drug users may reduce consumption^10^. However, there is an undeniable need for more comprehensive strategies to fully understand the epidemic, including a strategy that focuses on potential differences among demographic groups, standardization of the guidelines for the formulation of prescription opioids, and appropriate training of medical personnel^10^, especially since an opioid epidemic could be a key driver of infectious disease outbreaks such as HIV, hepatitis C virus, and endocarditis^32^.

## Methods

### Data sources and demographic analysis

Data were provided by the Ohio Department of Health and included 20,938 unintentional deaths by drug overdose from January 1^st^2010, to December 31^st^, 2017 in Ohio. This dataset was filtered to include only deaths caused by prescription opioid overdose (International Classification of Diseases, 10th Revision (ICD-10) cause of death codes: T40.2, T40.4, T40.6) among the majority racial groups (white and black population) aged 20–64 years (more than 95% of cases were aged 20–64) to focus analysis on adults of working age. As a result, 11,790 deaths by unintentional prescription opioid overdose were included in the analysis. The dataset also included information about gender, race, and age of the deceased individuals. Cases were geolocated by zip code of residency using the American Community Survey conducted by the United States Census Bureau^33^.

Estimated population sizes were retrieved from the United States Census Bureau American FactFinder portal^33^ by race, gender, and age group for each year from 2010 to 2017 and aggregated by zip code. Specifically, population size for each year by zip code was determined using projections made from 2010 Census Bureau data to estimate the number of males and females for white and black populations across five-year age groups from age 20 to 64 years. Since population density is reported yearly, population by month and trimester were estimated using a non-parametric regression spline interpolation. Aggregated deaths by race, gender, and five-year period were merged with data on population density to compute opioid overdose death rates per 100,000 inhabitants in each demographic category. Population sizes, number of deaths, and death rates were also computed for each zip code and year for use in the spatial and spatiotemporal analyses.

Identification of high-risk groups was conducted using opioid overdose mortality rates by race and gender. Prescription opioid death rates were estimated using Poisson regression analysis to quantify the annual percentage change for each race-gender group, and to identify the group with the highest increase in death rate during the period of analysis. Demographic profiles of death rates and number of deaths by race, gender, and age group were used to determine which groups were most impacted by prescription opioid overdose.

### Spatial clustering analysis of the opioid overdose epidemic

The spatial distribution of prescription opioid overdose deaths was analyzed using a spatial scan statistical analysis of data from 2010 to 2017, implemented in the SaTScan software^34^. We used scan statistics to identify geographical locations where the number of prescription opioid overdose deaths was higher than expected under the null hypothesis of a random spatial distribution of the deaths across the state. We refer to clusters of prescription opioid overdose deaths as hotspots. We analyzed death counts (from 2010 to 2017) using the SaTScan Poisson model with the size of the population at risk by location included as an offset. Briefly, identification of hotspots using the Poisson model implemented in SaTScan is achieved by testing each potential cluster against the null hypothesis that the distribution of cases was proportional to the population size (no clustering) using likelihood ratio and t-tests^34^. A hotspot was identified if the p-value was less than 0.05 and the grouping contained more than three zip code locations.

### Space and space-time relative risk estimation

Spatial and spatiotemporal RR estimation of deaths caused by prescription opioid overdose at the zip code level were conducted using Bayesian models that were fit using an integrated nested Laplace approximation (INLA) implemented in the R-INLA software package^35^. INLA is a computational algorithm designed to approximate complex integrals involved in posterior distributions by using a latent Gaussian model approximation^13^. The number of deaths by prescription opioid overdose was modeled using a zero-inflated Poisson regression to accommodate excess zero counts in sparse area-specific data^36^ in the context of a Besag-York-Mollie (BYM) model^37^. The BYM model used incorporated spatially correlated random effects that were modeled using a conditionally autoregressive structure (i.e., neighboring zip codes have correlated random effects). A second unstructured random effect was included in the model, and its variance component was modeled with a diffuse gamma prior distribution. Spatial analysis was conducted by zip code on the total number of deaths from 2010 to 2017, while spatiotemporal analysis was conducted by zip code on monthly data from 2010 to 2017.

To identify areas with high risk of opioid overdose mortality rates, we computed the prevalence and average RR in 2017, and temporal changes in RR by zip code within hotspots and non-hotspots. The temporal change in RR was defined as the percent difference between the average RR for the last semester of 2017 and the first semester of 2010. Finally, smoothed surfaces of the estimated RR and temporal change in RR were mapped along with the identified hotspots. Maps were created using ArcGIS by Esri version 10.5 (http://www.esri.com)^38^, and basemaps were obtained from ESRI and National Geographic available at ArcGIS Online basemaps^39^.

### Temporal trend analysis

Temporal trends of overdose death rates were identified using Bayesian interrupted time series analysis implemented in the CausalIimpact package in R^40^. In short, the analysis quantifies significant changing trends of the opioid overdose death rate (over a 12-month period) from the death rate expected in the absence of any disturbance of the opioid epidemic. Disturbances are assumed to be caused by external events that could positively (or negatively) influence death rates of prescription opioid overdoses over time (e.g., new government policies for reducing opioids prescriptions, new drugs available in the market).

Using this analysis, we modeled death rates for Ohio as a time series of monthly periods to estimate the monthly percent difference between the observed period and the (unobservable) change of death rates that would have occurred under the absence of any disturbance (called a counterfactual)^40^. The algorithm uses three sources of information to construct the counterfactual. The first source was a control time series given by the Ohio population at risk (aged 20–64) for each month, and the analysis assumes that in the absence of a positive or negative disturbance, overdoses are proportional to the population at risk. Second, the algorithm uses the death rates before a given time to estimate the alternative outcome without the effect of the disturbance. Finally, prior distributions of the outcome are used to include prior knowledge about the model using Bayesian regression. The three sources of information are combined using a state-space time series model. Death rates under the counterfactual model are computed from their posterior distributions, and the difference between the monthly percent change in the observed and counterfactual death rates are used to identify significant trends of the opioid epidemic.

Using this method, we simulated changes in the trajectory of the epidemic each month from January 2011 to December 2016 to identify the periods when the most significant changes occurred. Data from a period of 12 months before and 12 months after were used to estimate the observed and counterfactual percent differences in death rates of the epidemic. Then, estimated p-values for each month were grouped by trimesters to avoid short term variations, and non-contiguous periods, with p-values less than 0.005 selected to represent significant temporal trends in the epidemic^41^.

A video was generated to illustrate the spatio-temporal dynamics of the opioid overdose cases (Supplementary Video 1). Maps in the video were created using the open source framework Mapbox GL JS v 1.3.1 (https://www.mapbox.com/)^42^, and basemaps were obtained from OpenStreetMap (https://www.openstreetmap.org/#map=5/38.007/−95.844). Plot charts in the video were created using d3.js. JavaScript library (https://d3js.org/). To learn more, visit https://www.mapbox.com/about/maps/ and http://www.openstreetmap.org/copyright. Data extracted from OpenStreetMap after September 2012 is licensed on terms of the Open Database License, “ODbL” 1.0, previously it was licensed CC-BY-SA 2.0.

### Ethic statement

All data used in this study were in accordance with the ethical standards, following protocols for secondary data analysis, and with the approval of the Institutional Review Board (IRB) University of Cincinnati number CR01_2017–7637, and the Ohio Department of Health.

## Supplementary information

Supplementary Video.

Supplementary Data.

## Data Availability

The datasets generated and analyzed during the current study are not publicly available due to privacy restrictions of sensitive data. However, unidentified datasets are available from the corresponding author on reasonable request and with permission of the Ohio Department of Public Health.

## Code availability

Code for all analyses are available within the article and its Supplementary Information.
