# Supplementary material for: Epidemiological and geospatial profile of the prescription opioid crisis in Ohio, United States
Source: Sci Rep. 2020 Mar 9;10:4341. doi: 10.1038/s41598-020-61281-y (PMC7063043; doi:10.1038/s41598-020-61281-y)
Supplement: Supplementary file 2 — Supplementary Data. [file 41598_2020_61281_MOESM2_ESM.pdf]

Supplementary materials for:

Epidemiological and geospatial profile of the prescription opioid crisis in Ohio, United States

Andres Hernandez[1,2], Adam J. Branscum[3], Jingjing Li[4], Neil MacKinnon[5], Ana L. Hincapie[5], Diego F. Cuadros[1,2*]

[1]*Department of Geography and Geographic Information Science, University of Cincinnati, Cincinnati, USA*

[2]*Health Geography and Disease Modeling Laboratory, University of Cincinnati, Cincinnati, USA*

[3]*Department of Biostatistics, College of Public Health and Human Sciences, Oregon State University, Corvallis, USA*

[4]*Urban Health Collaborative, Dornsife School of Public Health, Drexel University, Philadelphia, Pennsylvania, USA*

[5]*James L. Winkle College of Pharmacy, University of Cincinnati, Cincinnati, Ohio, USA*

*Reprints or correspondence: Diego F. Cuadros, PhD, E-mail: diego.cuadros@uc.edu. Department of Geography and Geographic Information Science, University of Cincinnati, Cincinnati, OH, 45221. Telephone: (513) 556-3423. Fax: (513) 556-3370.

SUPPLEMENTARY FIGURES

Supplementary Figure 1. Opioid overdose mortality cases by county

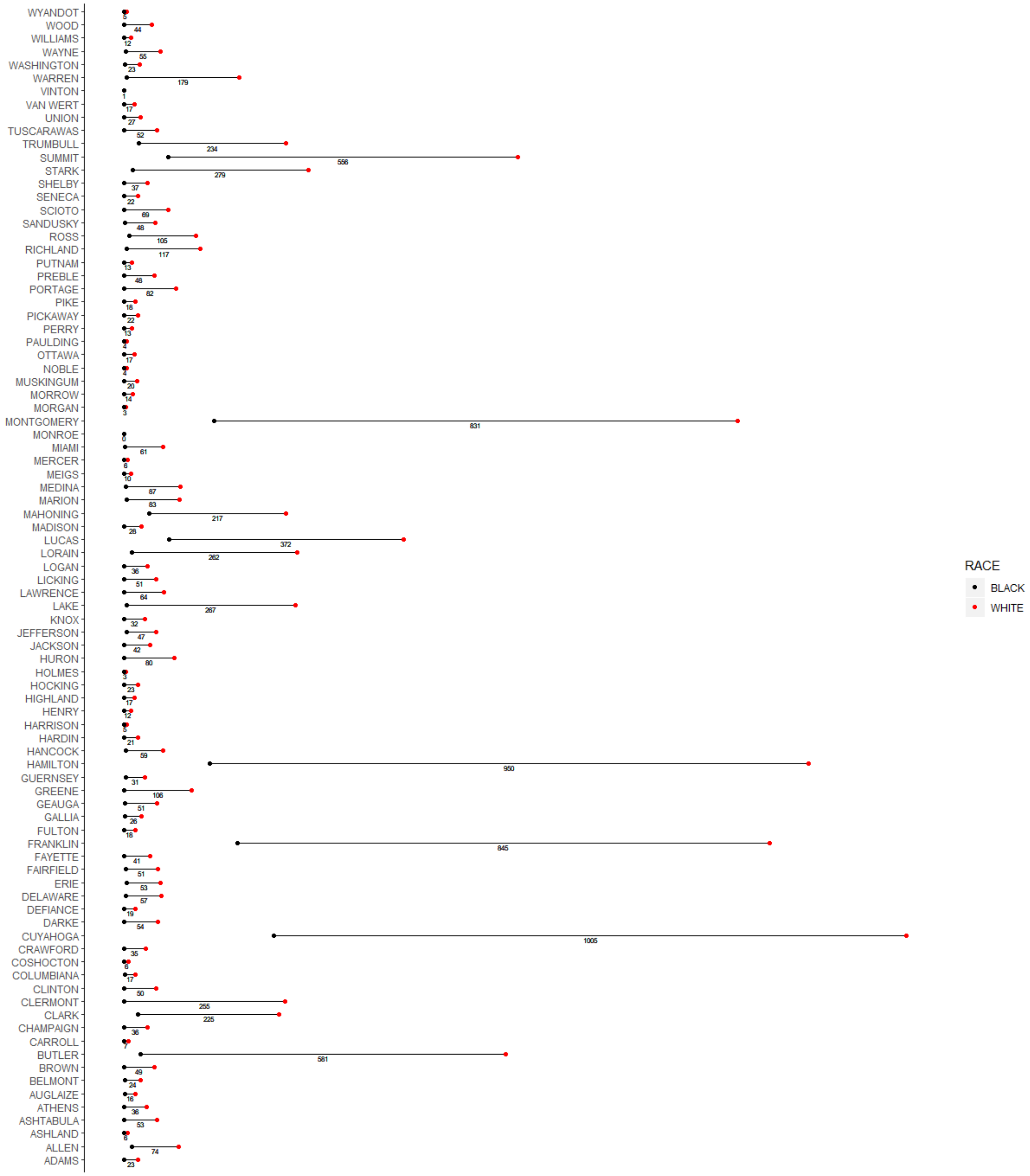

SUPPLEMENTARY TABLES

Supplementary Table 1. Detailed results temporal trending causal effect estimation.

PERIOD	Actual Death Rates Growth Trends	Predicted Death Rates Growth Trends	Absolute Effects	Relative Effects	Actual Cumulative Death Rates Growth Trends	Predicted Cumulative Death Rates Growth Trends	Absolute Cumulative Effects	Sig.[1]
2010-12	-0.173	-0.150	-0.023	0.155	-1.903	-1.647	-0.256	0.320
2011-01	-0.231	-0.165	-0.065	0.395	-2.539	-1.820	-0.719	0.076
2011-02	-0.250	-0.125	-0.125	0.997	-2.751	-1.378	-1.373	0.004
2011-03	-0.274	-0.105	-0.169	1.611	-3.015	-1.154	-1.860	**0.001**
2011-04	-0.268	-0.089	-0.179	2.019	-2.949	-0.977	-1.972	**0.001**
2011-05	-0.284	-0.065	-0.219	3.365	-3.121	-0.715	-2.406	**0.001**
2011-06	-0.297	-0.090	-0.207	2.302	-3.271	-0.991	-2.280	**0.001**
2011-07	-0.303	-0.086	-0.216	2.505	-3.332	-0.950	-2.381	**0.001**
2011-08	-0.325	-0.100	-0.225	2.253	-3.571	-1.098	-2.473	**0.001**
2011-09	-0.310	-0.089	-0.221	2.483	-3.409	-0.979	-2.430	**0.001**
2011-10	-0.293	-0.105	-0.188	1.795	-3.225	-1.154	-2.071	**0.002**
2011-11	-0.242	-0.206	-0.036	0.175	-2.665	-2.268	-0.397	0.363
2011-12	-0.183	-0.325	0.143	-0.438	-2.008	-3.577	1.568	0.192
2012-01	-0.179	-0.357	0.178	-0.498	-1.971	-3.931	1.959	0.181
2012-02	-0.176	-0.347	0.171	-0.493	-1.933	-3.816	1.883	0.202
2012-03	-0.170	-0.288	0.118	-0.410	-1.871	-3.171	1.300	0.280
2012-04	-0.162	-0.274	0.113	-0.410	-1.779	-3.018	1.238	0.300
2012-05	-0.150	-0.315	0.165	-0.525	-1.648	-3.468	1.820	0.192
2012-06	-0.162	-0.179	0.017	-0.093	-1.782	-1.965	0.182	0.467
2012-07	-0.150	-0.177	0.027	-0.154	-1.645	-1.945	0.300	0.448
2012-08	-0.176	0.040	-0.217	-5.403	-1.941	0.441	-2.382	0.123
2012-09	-0.162	0.004	-0.167	-37.983	-1.786	0.048	-1.834	0.175
2012-10	-0.136	-0.008	-0.128	15.933	-1.497	-0.088	-1.409	0.230

2012-11	-0.128	-0.035	-0.093	2.690	-1.404	-0.380	-1.024	0.295
2012-12	-0.123	-0.119	-0.004	0.035	-1.352	-1.306	-0.045	0.492
2013-01	-0.120	-0.218	0.098	-0.451	-1.315	-2.393	1.078	0.121
2013-02	-0.134	-0.129	-0.005	0.040	-1.475	-1.418	-0.057	0.469
2013-03	-0.086	-0.105	0.019	-0.179	-0.949	-1.157	0.208	0.419
2013-04	-0.006	-0.103	0.097	-0.939	-0.069	-1.135	1.066	0.166
2013-05	0.018	-0.092	0.111	-1.199	0.202	-1.015	1.217	0.137
2013-06	0.032	-0.137	0.169	-1.235	0.355	-1.507	1.861	0.039
2013-07	0.074	-0.090	0.164	-1.822	0.815	-0.992	1.807	0.039
2013-08	0.121	-0.158	0.279	-1.768	1.332	-1.733	3.065	**0.005**
2013-09	0.180	-0.036	0.216	-6.060	1.981	-0.392	2.373	0.007
2013-10	0.222	-0.080	0.302	-3.782	2.440	-0.877	3.317	**0.001**
2013-11	0.275	-0.121	0.397	-3.268	3.030	-1.336	4.367	**0.001**
2013-12	0.327	-0.133	0.459	-3.466	3.595	-1.458	5.053	**0.001**
2014-01	0.390	-0.114	0.504	-4.413	4.291	-1.257	5.548	**0.001**
2014-02	0.415	0.235	0.180	0.767	4.569	2.585	1.984	0.088
2014-03	0.419	0.744	-0.325	-0.437	4.605	8.182	-3.576	0.035
2014-04	0.479	0.796	-0.317	-0.399	5.267	8.759	-3.492	0.045
2014-05	0.567	0.670	-0.103	-0.154	6.235	7.369	-1.134	0.308
2014-06	0.609	0.787	-0.177	-0.226	6.700	8.652	-1.952	0.196
2014-07	0.637	0.813	-0.176	-0.216	7.007	8.943	-1.935	0.190
2014-08	0.654	0.988	-0.335	-0.339	7.191	10.872	-3.682	0.048
2014-09	0.692	0.919	-0.227	-0.247	7.615	10.108	-2.493	0.124
2014-10	0.714	0.986	-0.272	-0.275	7.857	10.845	-2.988	0.077
2014-11	0.749	0.945	-0.196	-0.208	8.240	10.400	-2.159	0.149
2014-12	0.785	0.972	-0.187	-0.193	8.633	10.692	-2.059	0.164
2015-01	0.807	1.003	-0.196	-0.195	8.877	11.028	-2.151	0.120
2015-02	0.848	0.934	-0.086	-0.092	9.330	10.278	-0.948	0.267
2015-03	0.871	1.112	-0.241	-0.216	9.582	12.229	-2.647	0.046

2015-04	0.903	1.419	-0.517	-0.364	9.931	15.613	-5.682	**0.001**
2015-05	0.944	1.369	-0.425	-0.311	10.383	15.061	-4.678	**0.001**
2015-06	0.993	1.126	-0.133	-0.118	10.920	12.385	-1.466	0.131
2015-07	1.025	1.058	-0.034	-0.032	11.270	11.643	-0.372	0.359
2015-08	1.076	0.992	0.083	0.084	11.831	10.915	0.916	0.172
2015-09	1.123	0.989	0.134	0.135	12.357	10.884	1.473	0.061
2015-10	1.180	0.949	0.231	0.243	12.979	10.443	2.537	**0.001**
2015-11	1.221	1.005	0.215	0.214	13.427	11.058	2.369	0.004
2015-12	1.258	1.000	0.258	0.258	13.840	11.004	2.836	**0.002**
2016-01	1.294	1.073	0.221	0.206	14.233	11.805	2.428	**0.003**
2016-02	1.359	1.125	0.233	0.207	14.946	12.380	2.566	**0.002**
2016-03	1.401	1.257	0.144	0.114	15.411	13.829	1.582	0.054
2016-04	1.431	1.359	0.072	0.053	15.743	14.951	0.792	0.212
2016-05	1.494	1.450	0.044	0.031	16.436	15.947	0.489	0.301
2016-06	1.556	1.462	0.094	0.064	17.121	16.085	1.036	0.131
2016-07	1.579	1.494	0.085	0.057	17.368	16.433	0.935	0.149
2016-08	1.601	1.520	0.080	0.053	17.609	16.724	0.885	0.163
2016-09	1.590	1.599	-0.008	-0.005	17.495	17.585	-0.091	0.465
2016-10	1.603	1.626	-0.023	-0.014	17.636	17.888	-0.251	0.403
2016-11	1.605	1.613	-0.008	-0.005	17.655	17.740	-0.085	0.451
2016-12	1.601	1.671	-0.070	-0.042	17.611	18.381	-0.770	0.175

1. Bold-face numbers indicate significance values (p-values < 0.005)

Epidemiological and geospatial profile of the prescription opioid crisis in Ohio, United States

*Andres Hernandez, Adam J. Branscum, Jingjing Li, Neil MacKinnon, Ana L. Hincapie, Diego F. Cuadros**

Demographic analysis

```
library(ggplot2)
OpioidsCases <- read.csv("./Datasets/Compiled_located.csv")
zip_clipped <- c()
for (element in OpioidsCases$Zip_of_death) {
  zip_clipped[length(zip_clipped)+1] <- substr(element, 1, 5)
}
OpioidsCases$zip_death_c <- zip_clipped
zip_clipped <- c()
for (element in OpioidsCases$Zip_of_residence) {
  zip_clipped[length(zip_clipped)+1] <- substr(element, 1, 5)
}
OpioidsCases$zip_residence_c <- zip_clipped
OpioidFiltered <- subset(OpioidsCases, Prescription_opiates == "Yes",
                         # OpioidFiltered <- subset(OpioidsCases, Opiates_all == "Yes",
                         select = c(
                           "Year_of_death",
                           "Month_of_death",
                           "Day_of_death",
                           "Age_In_Years",
                           "Race",
                           "Sex",
                           "Occupation",
                           "Marital_status",
                           "zip_death_c",
                           "zip_residence_c",
                           "Multiple_cause_of_death",
                           "Cause_of_death1",
                           "Cause_of_death2",
                           "Cause_of_death3",
                           "Cause_of_death4",
                           "How_injured",
                           "Other_condition",
                           "Alcohol_all_types",
                           "Heroin",
                           "Benzodiazepines",
                           "Methadone",
                           "Cocaine",
                           "Hallucinogens",
                           "Barbituates",
                           "Alcohol_Ethanol",
                           "Methanol",
                           "Alcohol_unspecified",
                           "Fentanyl_and_Analogues",
                           "Carfentanil"
                         ))

TEMPFILTER <- rbind (
OpioidFiltered[OpioidFiltered$Race=="WHITE",],
OpioidFiltered[OpioidFiltered$Race=="BLACK",]
)

OpioidFiltered <- TEMPFILTER
OpioidFiltered$Year_Month <- OpioidFiltered$Year_of_death*100+OpioidFiltered$Month_of_death
```

```
OpioidAgeAdj <- OpioidFiltered[OpioidFiltered$Age_In_Years > 19 & OpioidFiltered$Age_In_Years <
65, ]

OpioidDeathsZipCodeYear <- as.data.frame.matrix(table(OpioidAgeAdj$zip_residence_c, OpioidAgeAdj
$Year_Month))
OpioidDeathsZipCodeYear$Total <- rowSums(OpioidDeathsZipCodeYear)
OpioidDeathsZipCodeYear$ZipCodes <- rownames(OpioidDeathsZipCodeYear)

# Population File Management
Population_2010 <- read.csv("./Datasets/POP_ZIP_AGG_OH_2010.csv", sep = ",")
Population_20112017 <- read.csv("./Datasets/PopZipCode2011-2017.csv", sep = ",")

pop.whitemales2064_2010 <- apply(Population_2010[,11:22], 1, sum)
pop.whitefemales2064_2010 <- apply(Population_2010[,35:46], 1, sum)
pop.blackmales2064_2010 <- apply(Population_2010[,59:70], 1, sum)
pop.blackfemales2064_2010 <- apply(Population_2010[,83:94], 1, sum)

pop.whitemales2064_20117 <- apply(Population_20112017[,12:17], 1, sum)
pop.whitefemales2064_20117 <- apply(Population_20112017[,27:32], 1, sum)
pop.blackmales2064_20117 <- apply(Population_20112017[,43:48], 1, sum)
pop.blackfemales2064_20117 <- apply(Population_20112017[,58:63], 1, sum)

adjusted.pop_2010 <- apply(cbind(
  pop.whitemales2064_2010,
  pop.whitefemales2064_2010,
  pop.blackmales2064_2010,
  pop.blackfemales2064_2010
), 1, sum)

adjusted.pop.zipcode_2010 <- data.frame(cbind(Population_2010[,1], 2010, adjusted.pop_2010))
names(adjusted.pop.zipcode_2010) <- c("zipcode", "year", "pop")

adjusted.pop_20117 <- apply(cbind(
  pop.whitemales2064_20117,
  pop.whitefemales2064_20117,
  pop.blackmales2064_20117,
  pop.blackfemales2064_20117
), 1, sum)

adjusted.pop.zipcode_20117 <- cbind(Population_20112017[,1], Population_20112017[67], adjusted.p
op_20117)
names(adjusted.pop.zipcode_20117) <- c("zipcode", "year", "pop")

adjusted.pop.zipcode <- rbind(
  adjusted.pop.zipcode_2010,
  adjusted.pop.zipcode_20117
)

# Adjust by age Population
TempPop_2010 <- as.data.frame(subset(adjusted.pop.zipcode, year ==2010, c("zipcode", "pop")))
TempPop_2011 <- as.data.frame(subset(adjusted.pop.zipcode, year ==2011, c("pop")))
TempPop_2012 <- as.data.frame(subset(adjusted.pop.zipcode, year ==2012, c("pop")))
TempPop_2013 <- as.data.frame(subset(adjusted.pop.zipcode, year ==2013, c("pop")))
TempPop_2014 <- as.data.frame(subset(adjusted.pop.zipcode, year ==2014, c("pop"))[-1,])
```

```
TempPop_2015 <- as.data.frame(subset(adjusted.pop.zipcode, year ==2015, c("pop"))[-1,])
TempPop_2016 <- as.data.frame(subset(adjusted.pop.zipcode, year ==2016, c("pop"))[-1,])
TempPop_2017 <- as.data.frame(subset(adjusted.pop.zipcode, year ==2017, c("pop")))

TempPop <- cbind(TempPop_2010, TempPop_2011, TempPop_2012, TempPop_2013, TempPop_2014, TempPop_2
015, TempPop_2016, TempPop_2017)
names(TempPop) <- c("ZipCode", "A2010", "A2011", "A2012", "A2013", "A2014", "A2015", "A2016", "A
2017")

linearmodel <- function(x){
  df <- data.frame(Years = seq(0, 84, 12))
  df$Pop <- x[2:9]
  df$Pop[which(df$Pop==0)] <- 1
  model1 <- lm(log(Pop) ~ Years, data = df)
  predicted <- exp(predict(model1, newdata = data.frame(Years = c(0:95))))
  return(predicted)
}

splinemodel <- function(x) {
  model1 <- smooth.spline(seq(0, 84, 12), x[2:9])
  return(predict(model1, 1:96)$y)
}

predictedPopDf <- data.frame(t(apply(TempPop, 1, splinemodel)))
predictedPopDf$ZipCodes <- TempPop$ZipCode

TempOhioZipCodeYear <- OpioidDeathsZipCodeYear
TempOhioZipCodeYear2 <- matrix(0,1,98)

for(i in as.numeric(predictedPopDf$ZipCodes)){
  if(!substring(as.character(i),1,1) == "*") {
    if(!i %in% TempOhioZipCodeYear$ZipCode){
      # print(i)
      temp <- cbind(matrix(0,1,97), i)
      # print(temp)
      # print("+++++")
      TempOhioZipCodeYear2 <- rbind(TempOhioZipCodeYear2, temp)
    } else {
      temp <- matrix(as.numeric(TempOhioZipCodeYear[TempOhioZipCodeYear$ZipCodes==i, ]), 1, 98)
      # print(temp)
      # print("*****")
      TempOhioZipCodeYear2 <- rbind(TempOhioZipCodeYear2, temp)
    }
  }
}

TempOhioZipCodeYear2 <- as.data.frame(TempOhioZipCodeYear2)
TempOhioZipCodeYear2 <- TempOhioZipCodeYear2[-1,]

names(TempOhioZipCodeYear2) <- names(OpioidDeathsZipCodeYear)
rownames(TempOhioZipCodeYear2) <- TempOhioZipCodeYear2$ZipCodes

PopDeaths <- merge(OpioidDeathsZipCodeYear[,-97], predictedPopDf, by = "ZipCodes")
aggOpioidsDeath <- data.frame(ZipCodes=PopDeaths$ZipCodes)
```

```
aggOpioidsPop <- data.frame(ZipCodes=PopDeaths$ZipCodes)

OpioidsDeathsZipCode <- data.frame(zipcodes = aggOpioidsDeath$ZipCodes)
OpioidsDeathsZipCode$Deaths <- rowSums(aggOpioidsDeath[,-1])

Ind_cases <- OpioidAgeAdj[1,]
Ind_cases <- Ind_cases[c(-1),]

k <- 1
for(i in OpioidAgeAdj$zip_residence_c){
  # print(i)
  if(i %in% OpioidsDeathsZipCode$zipcodes) {
    # print("match")
    # print(i)
    # print(OpioidAgeAdj[k,])
    Ind_cases <- rbind(Ind_cases, OpioidAgeAdj[k,])
  }
  k <- k+1
}

Ind_cases_Filter <- Ind_cases[Ind_cases$Race=="BLACK",]
brakes <- c(20,25,30,35,40,45,50,55,60,65)
DemographicPyramid <- data.frame(agegroup = cut(Ind_cases_Filter$Age_In_Years,breaks=brakes,incl
ude.lowest=TRUE, right = FALSE))
DemographicPyramid$sex <- Ind_cases_Filter$Sex
DemographicPyramid$year <- Ind_cases_Filter$Year_of_death

demplotdata <- data.frame( table(DemographicPyramid$agegroup, DemographicPyramid$sex)[,1] )
demplotdata$gender <- "F"
demplotdata2 <- data.frame( table(DemographicPyramid$agegroup, DemographicPyramid$sex)[,2] ) * -
1
demplotdata2$gender <- "M"
demplotdataTotal <- rbind(demplotdata, demplotdata2)
names(demplotdataTotal) <- c("CASES", "GENDER")
demplotdataTotal$AGE_GROUP <-
  c("20-24", "25-29", "30-34", "35-39", "40-44", "45-49", "50-54", "55-59", "60-64",
    "20-24", "25-29", "30-34", "35-39", "40-44", "45-49", "50-54", "55-59", "60-64")

#input_dataset$AGE_GROUP = factor(input_dataset$AGE_GROUP,levels(input_dataset$AGE_GROUP)[c(1,3:
17)])
input_dataset <-  demplotdataTotal

labels <- input_dataset
labelsF <- labels[labels$GENDER == 'F',]
labelsM <- labels[labels$GENDER == 'M',]

# Limits for black population
lower_limit <- 160
upper_limit <- 160
true_limit <- max (lower_limit, upper_limit)
seq_limit <- seq(-1*true_limit, true_limit, round(true_limit/10))
seq_false <- abs(seq_limit)

pyramidGH2 <- ggplot(labels, aes(x = AGE_GROUP, y = CASES, fill = GENDER)) +
```

```
geom_bar(data = labelsF, stat = "identity") +
geom_bar(data = labelsM, stat = "identity") +
xlab("") +
ylab("") +
scale_y_continuous(
  limits = c(-1*true_limit, true_limit),
  breaks = seq(-150, 150, 50),
  labels = c("150", "100","50","0","50","100", "150")
) +
scale_fill_manual(values=alpha(c("#ff00e1", "#2500ff"),0.5)) +
geom_bar(data = labelsF, colour="black", stat = "identity", size=1) +
geom_bar(data = labelsM, colour="black", stat = "identity", size=1) +
geom_text(data=labelsF, aes(x = AGE_GROUP, y = CASES*1.1+10, label = CASES), size = 5, position
 = "identity", col=I("#ff00e1")) +
geom_text(data=labelsM, aes(x = AGE_GROUP, y = CASES*1.1-15, label = -1 * CASES), size = 5, posi
tion = position_stack(vjust = 0.1), col=I("#2500ff")) +

theme_minimal() +
theme(axis.text = element_text(size = 15, colour="black")) +
coord_flip()
pyramidGH2
```

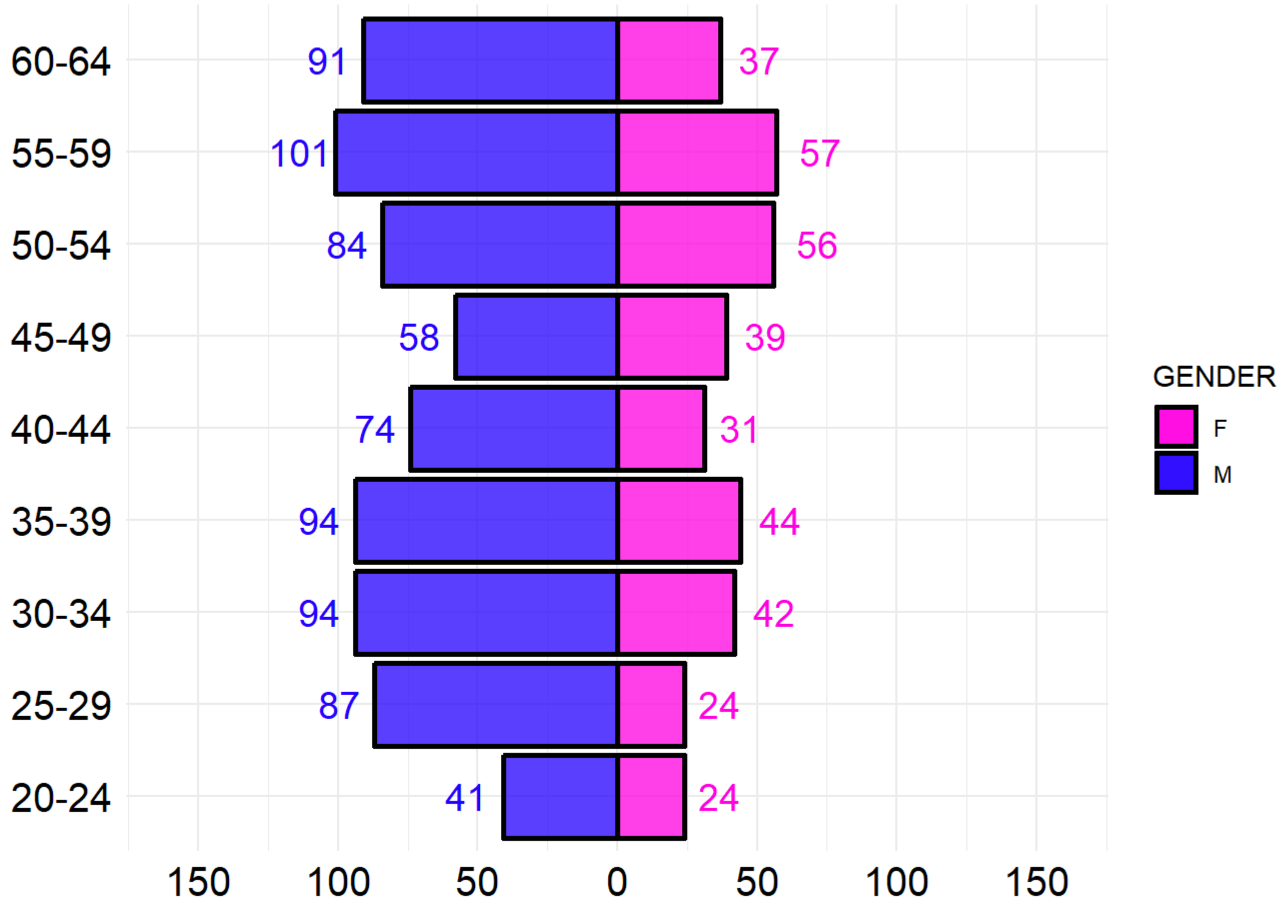

```
Ind_cases_Filter <- Ind_cases[Ind_cases$Race=="WHITE",]
brakes <- c(20,25,30,35,40,45,50,55,60,65)
DemographicPyramid <- data.frame(agegroup = cut(Ind_cases_Filter$Age_In_Years,breaks=brakes,incl
ude.lowest=TRUE, right = FALSE))
DemographicPyramid$sex <- Ind_cases_Filter$Sex
DemographicPyramid$year <- Ind_cases_Filter$Year_of_death

demplotdata <- data.frame( table(DemographicPyramid$agegroup, DemographicPyramid$sex)[,1] )
demplotdata$gender <- "F"
demplotdata2 <- data.frame( table(DemographicPyramid$agegroup, DemographicPyramid$sex)[,2] ) * -
1
demplotdata2$gender <- "M"
demplotdataTotal <- rbind(demplotdata, demplotdata2)
names(demplotdataTotal) <- c("CASES", "GENDER")
demplotdataTotal$AGE_GROUP <-
  c("20-24", "25-29", "30-34", "35-39", "40-44", "45-49", "50-54", "55-59", "60-64",
    "20-24", "25-29", "30-34", "35-39", "40-44", "45-49", "50-54", "55-59", "60-64")

#input_dataset$AGE_GROUP = factor(input_dataset$AGE_GROUP,levels(input_dataset$AGE_GROUP)[c(1,3:
17)])
input_dataset <-  demplotdataTotal

labels <- input_dataset
labelsF <- labels[labels$GENDER == 'F',]
labelsM <- labels[labels$GENDER == 'M',]

#White Population Plot
# Limits for white population
lower_limit <- 1600
upper_limit <- 1600

# Limits for black population
true_limit <- max (lower_limit, upper_limit)
seq_limit <- seq(-1*true_limit, true_limit, round(true_limit/10))
seq_false <- abs(seq_limit)


pyramidGH3 <- ggplot(labels, aes(x = AGE_GROUP, y = CASES, fill = GENDER)) +
geom_bar(data = labelsF, stat = "identity") +
geom_bar(data = labelsM, stat = "identity") +
xlab("") +
ylab("") +
scale_y_continuous(
  limits = c(-1*true_limit, true_limit),
  breaks = seq(-1500, 1500, 500),
  labels = c("1500", "1000","500","0","500","1000", "1500")
) +
scale_fill_manual(values=alpha(c("#ff00e1", "#2500ff"),0.5)) +
geom_bar(data = labelsF, colour="black", stat = "identity", size=1) +
geom_bar(data = labelsM, colour="black", stat = "identity", size=1) +
# geom_text(data=labelsF, aes(x = AGE_GROUP, y = CASES, label = CASES), size = 2.5, position = p
osition_stack(vjust = 10), col=I("#000000")) +
geom_text(data=labelsF, aes(x = AGE_GROUP, y = CASES*1.1+100, label = CASES), size = 5, position
```

```
= "identity", col=I("#ff00e1")) +
geom_text(data=labelsM, aes(x = AGE_GROUP, y = CASES*1.1-150, label = -1 * CASES), size = 5, pos
ition = position_stack(vjust = 0.1), col=I("#2500ff")) +
# geom_text(data=labelsF, aes(x = AGE_GROUP, y = CASES*1.1+10, label = CASES), size = 5, positio
n = "identity", col=I("#ff00e1")) +
# geom_text(data=labelsM, aes(x = AGE_GROUP, y = CASES*1.1-15, label = -1 * CASES), size = 5, po
sition = position_stack(vjust = 0.1), col=I("#2500ff")) +
theme_minimal() +
theme(axis.text = element_text(size = 15, colour="black")) +
# geom_text(data=labelsF, aes(x = AGE_GROUP, y = CASES*1.1+450, label = paste('(',RATES,')', sep
= "")), size = 3, position = "identity", col=I("#ff00e1")) +
# geom_text(data=labelsM, aes(x = AGE_GROUP, y = CASES*1.1-250, label = paste('(',RATES,')', sep
= "")), size = 3, position = position_stack(vjust = 0.1), col=I("#2500ff")) +
coord_flip()
pyramidGH3
```

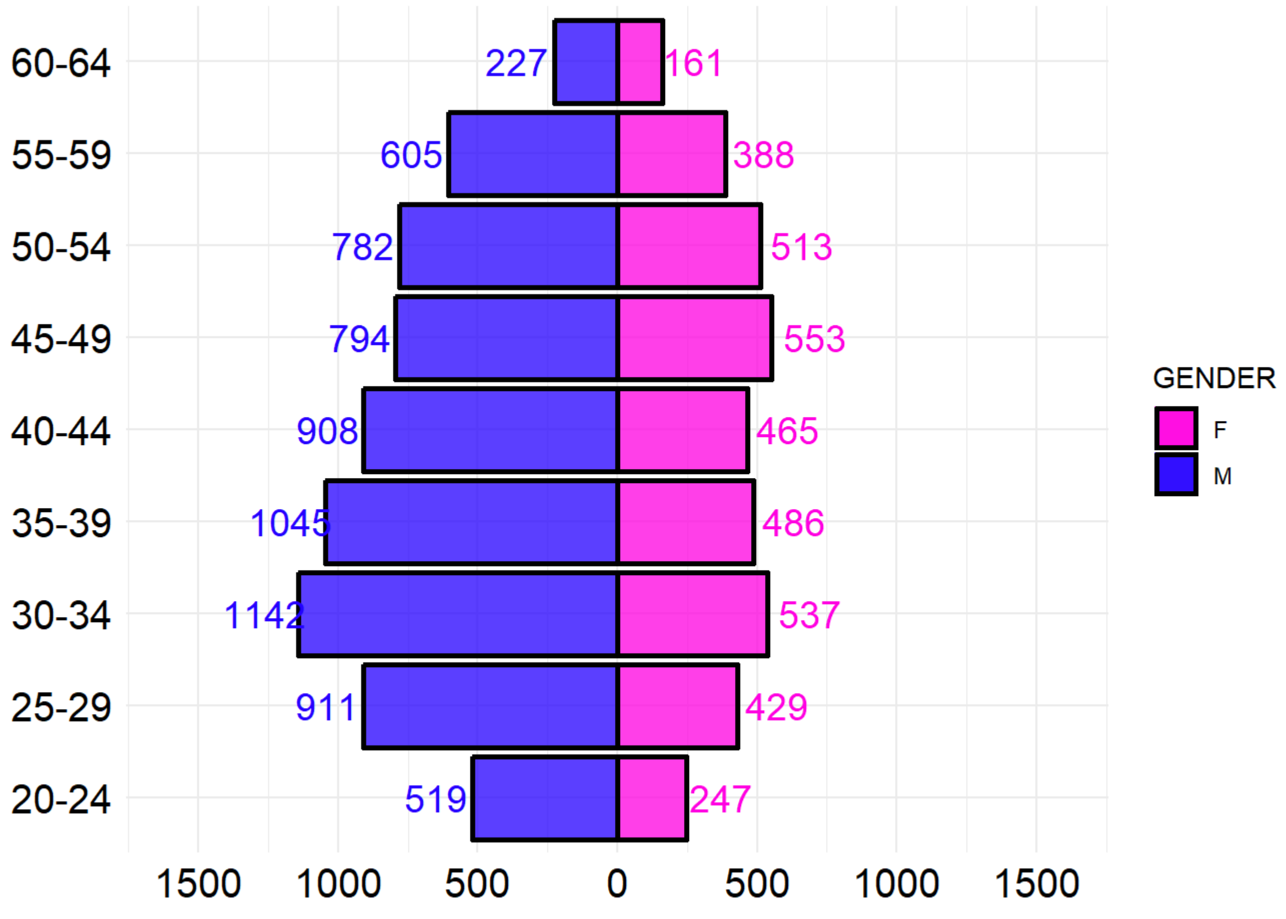

Space and space-time relative risk estimation

Relative Risk Spatial Analysis

```
library(sf)
library(spdep)
library(INLA)
library(SpatialEpi)
library(rgdal)
library(leaflet)

load("./Datasets/AggCases.RData")

# Create expected cases Matrix
CasesZipTime <- CasesZipTime_All
PopZipTime <- PopZipTime_All
casecounts <- data.frame(Cases = apply(CasesZipTime[,-1], 1, sum))
casecounts$ZipCode <- CasesZipTime$ZipCodes
E <- expected(population = PopZipTime[,33], cases = casecounts$Cases, n.strata = 1)

# Read shapefile
# ShapeFile <- readOGR("/home/andres/Desktop/OpioidsManuscript/Hernandez_EpidemiologicGeospatial
OpioidsOhio_Code/Datasets/OhioZipShape","OhioZipCodesWSG1984")
ShapeFile <- readOGR("C:\\Users\\Andres\\Desktop\\OpioidsManuscript\\Hernandez_EpidemiologicGeos
patialOpioidsOhio_Code\\Datasets\\OhioZipShape","OhioZipCodesWSG1984")
FilteredShapeFile <- ShapeFile[ShapeFile$ZCTA5CE10 %in% CasesZipTime$ZipCodes,]
RenamedFilteredShapeFile <- spChFIDs(FilteredShapeFile, as.character(FilteredShapeFile$ZCTA5CE1
0))

# Create neighbors matrix
matrixEpi <- data.frame(ZipCode = casecounts$ZipCode, Cases = casecounts$Cases, E = E, SMR = rou
nd(casecounts$Cases/E,3))

rownames(matrixEpi) <- matrixEpi$ZipCode
map <- SpatialPolygonsDataFrame(RenamedFilteredShapeFile, matrixEpi, match.ID = TRUE)

nb <- poly2nb(map) # create neighbors
nb2INLA("./Datasets/neighzip.adj", nb)
adj.matrix <- inla.read.graph(filename = "./Datasets/neighzip.adj")

# visualize Map
# Create INLA models
map$re_u <- 1:nrow(map@data)
map$re_v <- 1:nrow(map@data)

# Spatial
formula <- Cases ~ 1 +
  f( re_u,
     model = "bym",
     graph = adj.matrix,
     scale.model = TRUE,
     adjust.for.con.comp = TRUE ,
     hyper = list (
       prec.unstruct =
         list (
           prior = "loggamma",
           param = c (1 , 0.001)
```

```
          ),
        prec.spatial =
          list (
            prior = "loggamma",
            param = c (1 , 0.001)
          )
      )
  ) +
  f(
    re_v,
      model = "iid"
  )

res <- inla(
  formula,
  # family = "poisson",
  family = "zeroinflatedpoisson1",
  data = map@data,
  E = E,
  control.predictor = list(compute = TRUE)
)

map$RR <- res$summary.fitted.values[, "mean"]
map$LL <- res$summary.fitted.values[, "0.025quant"]
map$UL <- res$summary.fitted.values[, "0.975quant"]

map$RR[map$RR > 100] <- 100
map$RR[map$RR < -100] <- -100

map$LL[map$LL > 100] <- 100
map$LL[map$LL < -100] <- -100

map$UL[map$UL > 100] <- 100
map$UL[map$UL < -100] <- -100

# Mapping relative risk
mapPlot <- map

library(BAMMtools)
binsdrates <- getJenksBreaks(mapPlot$RR, 6, subset = NULL)
bins <- c(0, binsdrates, Inf)
pal <- colorBin("YlOrRd", domain = mapPlot$RR, bins = bins)

l <- leaflet(mapPlot) %>% addTiles()
l %>% addPolygons(color = "grey", weight = 1, fillColor = ~pal(RR), fillOpacity = 0.5) %>%
  addLegend(pal = pal, values = ~RR, opacity = 0.5, title = "Aggregated RR 2010-2017", position
 = "bottomright")

# writeOGR(obj=map, dsn="C:\\Users\\Andres\\Documents\\Andres\ Documents\\Summer2019\\Opioids",
 layer="opioids_spatial_W", driver="ESRI Shapefile")
```

```
## OGR data source with driver: ESRI Shapefile
## Source: "C:\Users\Andres\Desktop\OpioidsManuscript\Hernandez_EpidemiologicGeospatialOpioidsOh
io_Code\Datasets\OhioZipShape", layer: "OhioZipCodesWSG1984"
## with 1198 features
## It has 18 fields
## Integer64 fields read as strings:  OBJECTID OBJECTID_1
```

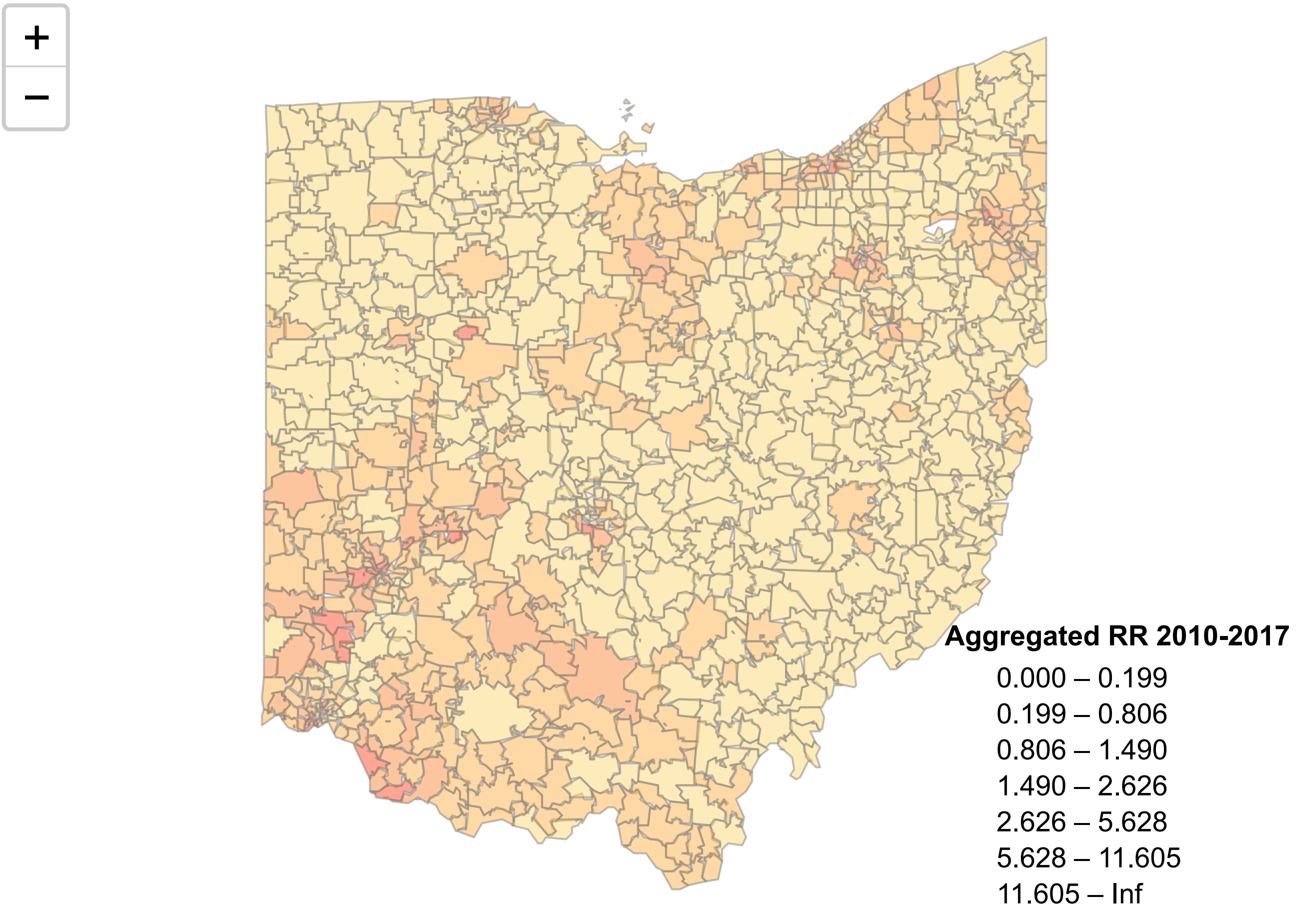

Prints use map data from Leaflet and OpenStreetMap and their data sources. To learn more, visit https://leafletjs.com/ and http://www.openstreetmap.org/copyright. Data extracted from OpenStreetMap after September 2012 is licensed on terms of the Open Database License, "ODbL" 1.0, previously it was licensed CC-BY-SA 2.0.

Relative Risk Spatio-temporal Analysis

```
# Spatio-temporal
order <- match (map$ZipCode, CasesZipTime_W$ZipCodes)
SpatioTemporalObjectCases.ord <- CasesZipTime_W[order, ]

order <- match (map$ZipCode, PopZipTime_W$ZipCodes)
SpatioTemporalObjectPop.ord <- PopZipTime_W[order, ]

SpatioTemporalObjectCases <- SpatioTemporalObjectCases.ord
SpatioTemporalObjectPop <- SpatioTemporalObjectPop.ord
TempSpatiotemporal <- matrix(0,1,6)

for(element in 1:length(colnames(SpatioTemporalObjectCases[,-1]))){
  temp <-
    as.data.frame(
      cbind(
        SpatioTemporalObjectCases[,1],
        1:length(SpatioTemporalObjectPop[,1]),
        rep(element, length(SpatioTemporalObjectPop[,1])),
        SpatioTemporalObjectCases[,element+1],
        SpatioTemporalObjectPop[,element+1],
        expected(population = SpatioTemporalObjectPop[,element+1], cases = SpatioTemporalObjectC
ases[,element+1], n.strata = 1)
      )
    )
  TempSpatiotemporal <- rbind(TempSpatiotemporal, temp)
}

TempSpatiotemporal <- as.data.frame(TempSpatiotemporal)
TempSpatiotemporal <- TempSpatiotemporal[-1,]
names(TempSpatiotemporal) <- c("zipcodes", "zipid", "year", "Y", "n", "E")
TempSpatiotemporal$zipid1 <- TempSpatiotemporal$zipid

adj.matrix <- inla.read.graph(filename = "./Datasets/neighzip.adj")
formula.par <- Y ~ f(zipid,model="bym",graph=adj.matrix, constr=TRUE) + f(zipid1,year,model="ii
d", constr=TRUE) + year

model.par <- inla(formula.par,family="poisson",data=TempSpatiotemporal,E=E,
                  control.predictor=list(compute=TRUE),
                  control.compute=list(dic=TRUE,cpo=TRUE),
                  num.threads = 20 )

m <- model.par$marginals.random[[1]][1:1197]
zeta.ST1 <- unlist(lapply(m,function(x)inla.emarginal(exp,x)))

SMR.cutoff<- c(0.0, 0.9, 1.0, 1.1,3)
xi.factor <- cut(zeta.ST1,breaks=SMR.cutoff,include.lowest=TRUE)
m <- model.par$summary.random[[2]][1:1197,2]
int.cut <- c(-0.008,-0.001,0,0.001,0.006)
int.factor <- cut(m,breaks=int.cut,include.lowest=TRUE)

TempFittedResults <- data.frame(
  ZipCode = TempSpatiotemporal$zipcodes[1:1197],
  FittedValues = model.par$summary.fitted.values[, "mean"][1:1197]
```

```
)

rownames(TempFittedResults) <- TempFittedResults$ZipCode
map <- SpatialPolygonsDataFrame(RenamedFilteredShapeFile, TempFittedResults, match.ID = TRUE)

map$Spatial <- xi.factor
map$Temporal <- int.factor
map$RR <- model.par$summary.fitted.values[, "mean"][1:1197]

firstSem <- rowMeans(
  cbind(
    model.par$summary.fitted.values[, "mean"][(1197*0+1):(1197*1)],
    model.par$summary.fitted.values[, "mean"][(1197*1+1):(1197*2)],
    model.par$summary.fitted.values[, "mean"][(1197*2+1):(1197*3)],
    model.par$summary.fitted.values[, "mean"][(1197*3+1):(1197*4)],
    model.par$summary.fitted.values[, "mean"][(1197*4+1):(1197*5)],
    model.par$summary.fitted.values[, "mean"][(1197*5+1):(1197*6)]
  )
)

lastSem <- rowMeans(
  cbind(
    model.par$summary.fitted.values[, "mean"][(1197*26+1):(1197*27)],
    model.par$summary.fitted.values[, "mean"][(1197*27+1):(1197*28)],
    model.par$summary.fitted.values[, "mean"][(1197*28+1):(1197*29)],
    model.par$summary.fitted.values[, "mean"][(1197*29+1):(1197*30)],
    model.par$summary.fitted.values[, "mean"][(1197*30+1):(1197*31)],
    model.par$summary.fitted.values[, "mean"][(1197*31+1):(1197*32)]
  )
)

map$RR2 <- (lastSem - firstSem)*100
map$RR3 <- (lastSem)

map$ZipCode[which(map$RR2>300, TRUE)]
map$Z <- map$RR2
map$Z[map$Z > 300] <- 300
map$Z[map$Z < -300] <- -300

risk2017 <- data.frame(zipcodes = map$ZipCode, risk2017 = lastSem)
mapPlot <- map

binsdrates <- getJenksBreaks(mapPlot$Z, 5, subset = NULL)
bins <- c(0, binsdrates, Inf)
pal <- colorBin("YlOrRd", domain = mapPlot$Z, bins = bins)

l <- leaflet(mapPlot) %>% addTiles()
l %>% addPolygons(color = "grey", weight = 1, fillColor = ~pal(Z), fillOpacity = 0.5) %>% addLeg
end(pal = pal, values = ~Z, opacity = 0.5, title = "Spatio-temporal RR change (2010-2017)", posi
tion = "bottomright")

# writeOGR(obj=map, dsn="C:\\Users\\Andres\\Documents\\Andres\ Documents\\Summer2019\\Opioids",
 layer="spatiotemporal_06112019_2", driver="ESRI Shapefile")
```

```
## [1] 45417 45403 44104
```

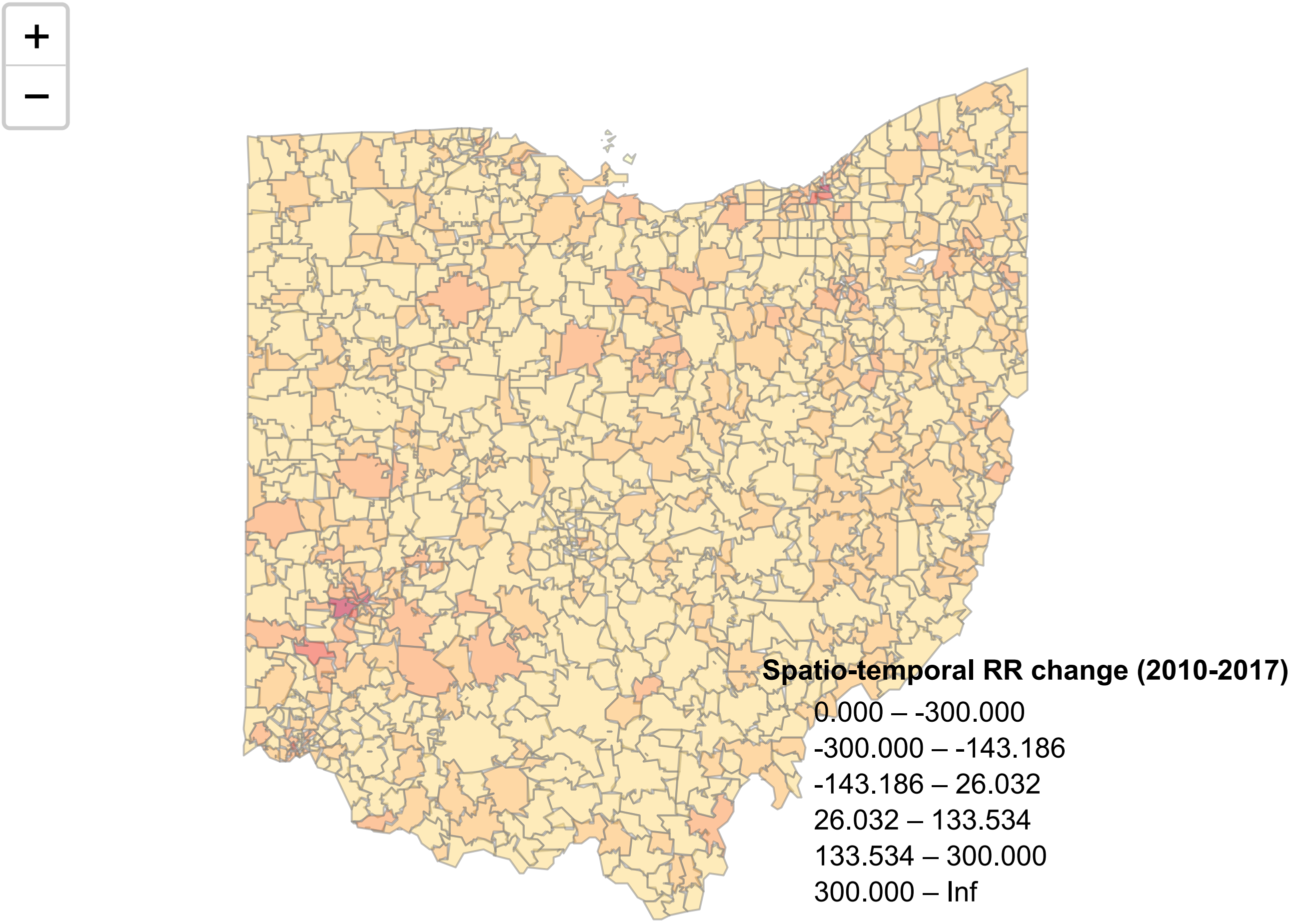

Prints use map data from Leaflet and OpenStreetMap and their data sources. To learn more, visit https://leafletjs.com/ and http://www.openstreetmap.org/copyright. Data extracted from OpenStreetMap after September 2012 is licensed on terms of the Open Database License, "ODbL" 1.0, previously it was licensed CC-BY-SA 2.0.

Temporal trend analysis

```
library(ggmap)
library(CausalImpact)

OpioidsCases <- read.csv("./Datasets/Compiled_located.csv") # Data availability upon request and
with authorization of the Ohio Department of Public Health

zip_clipped <- c()

for (element in OpioidsCases$Zip_of_death) {
  zip_clipped[length(zip_clipped)+1] <- substr(element, 1, 5)
}
OpioidsCases$zip_death_c <- zip_clipped

zip_clipped <- c()
for (element in OpioidsCases$Zip_of_residence) {
  zip_clipped[length(zip_clipped)+1] <- substr(element, 1, 5)
}
OpioidsCases$zip_residence_c <- zip_clipped

#Filter by prescription opioids
OpioidFiltered <- subset(OpioidsCases, Prescription_opiates == "Yes",
                       select = c(
                           "Year_of_death",
                           "Month_of_death",
                           "Day_of_death",
                           "Age_In_Years",
                           "Race",
                           "Sex",
                           "Occupation",
                           "Marital_status",
                           "zip_death_c",
                           "zip_residence_c",
                           "Multiple_cause_of_death",
                           "Cause_of_death1",
                           "Cause_of_death2",
                           "Cause_of_death3",
                           "Cause_of_death4",
                           "How_injured",
                           "Other_condition",
                           "Alcohol_all_types",
                           "Heroin",
                           "Benzodiazepines",
                           "Methadone",
                           "Cocaine",
                           "Hallucinogens",
                           "Barbituates",
                           "Alcohol_Ethanol",
                           "Methanol",
                           "Alcohol_unspecified",
                           "Fentanyl_and_Analogues",
                           "Carfentanil"
                         ))
```

```
TEMPFILTER <- rbind (
  OpioidFiltered[OpioidFiltered$Race=="WHITE",],
  OpioidFiltered[OpioidFiltered$Race=="BLACK",]
)

OpioidFiltered <- TEMPFILTER
OpioidFiltered$Year_Month <- OpioidFiltered$Year_of_death*100+OpioidFiltered$Month_of_death
OpioidAgeAdj <- OpioidFiltered[OpioidFiltered$Age_In_Years > 19 & OpioidFiltered$Age_In_Years <
65, ]

brakes <- c(20,25,30,35,40,45,50,55,60,65)
DemographicPyramid <- data.frame(agegroup = cut(OpioidAgeAdj$Age_In_Years,breaks=brakes,include.
lowest=TRUE, right = FALSE))
DemographicPyramid$sex <- OpioidAgeAdj$Sex
DemographicPyramid$year <- OpioidAgeAdj$Year_of_death

OpioidDeathsZipCodeYear <- as.data.frame.matrix(table(OpioidAgeAdj$zip_residence_c, OpioidAgeAdj
$Year_Month))
OpioidDeathsZipCodeYear$Total <- rowSums(OpioidDeathsZipCodeYear)
OpioidDeathsZipCodeYear$ZipCodes <- rownames(OpioidDeathsZipCodeYear)

#Population File Management
Population_2010 <- read.csv("./Datasets/POP_ZIP_AGG_OH_2010.csv", sep = ",")
Population_20112017 <- read.csv("./Datasets/PopZipCode2011-2017.csv", sep = ",")

pop.whitemales2064_2010 <- apply(Population_2010[,11:22], 1, sum)
pop.whitefemales2064_2010 <- apply(Population_2010[,35:46], 1, sum)
pop.blackmales2064_2010 <- apply(Population_2010[,59:70], 1, sum)
pop.blackfemales2064_2010 <- apply(Population_2010[,83:94], 1, sum)

pop.whitemales2064_20117 <- apply(Population_20112017[,12:17], 1, sum)
pop.whitefemales2064_20117 <- apply(Population_20112017[,27:32], 1, sum)
pop.blackmales2064_20117 <- apply(Population_20112017[,43:48], 1, sum)
pop.blackfemales2064_20117 <- apply(Population_20112017[,58:63], 1, sum)

adjusted.pop_2010 <- apply(cbind(
  pop.whitemales2064_2010,
  pop.whitefemales2064_2010
  ,
  pop.blackmales2064_2010,
  pop.blackfemales2064_2010
  ), 1, sum)

adjusted.pop.zipcode_2010 <- data.frame(cbind(Population_2010[,1], 2010, adjusted.pop_2010))
names(adjusted.pop.zipcode_2010) <- c("zipcode", "year", "pop")

adjusted.pop_20117 <- apply(cbind(
  pop.whitemales2064_20117,
  pop.whitefemales2064_20117
  ,
  pop.blackmales2064_20117,
  pop.blackfemales2064_20117
  ), 1, sum)
```

```
adjusted.pop.zipcode_20117 <- cbind(Population_20112017[,1], Population_20112017[67],
                                    adjusted.pop_20117
                                    )
names(adjusted.pop.zipcode_20117) <- c("zipcode", "year", "pop")

adjusted.pop.zipcode <- rbind(
  adjusted.pop.zipcode_2010,
  adjusted.pop.zipcode_20117
  )

#Adjust by age Population
TempPop_2010 <- as.data.frame(subset(adjusted.pop.zipcode, year ==2010, c("zipcode", "pop")))
TempPop_2011 <- as.data.frame(subset(adjusted.pop.zipcode, year ==2011, c("pop")))
TempPop_2012 <- as.data.frame(subset(adjusted.pop.zipcode, year ==2012, c("pop")))
TempPop_2013 <- as.data.frame(subset(adjusted.pop.zipcode, year ==2013, c("pop")))
TempPop_2014 <- as.data.frame(subset(adjusted.pop.zipcode, year ==2014, c("pop"))[-1,])
TempPop_2015 <- as.data.frame(subset(adjusted.pop.zipcode, year ==2015, c("pop"))[-1,])
TempPop_2016 <- as.data.frame(subset(adjusted.pop.zipcode, year ==2016, c("pop"))[-1,])
TempPop_2017 <- as.data.frame(subset(adjusted.pop.zipcode, year ==2017, c("pop")))

TempPop <- cbind(TempPop_2010, TempPop_2011, TempPop_2012, TempPop_2013, TempPop_2014, TempPop_2
015, TempPop_2016, TempPop_2017)
names(TempPop) <- c("ZipCode", "A2010", "A2011", "A2012", "A2013", "A2014", "A2015", "A2016", "A
2017")

linearmodel <- function(x){
  df <- data.frame(Years = seq(0, 84, 12))
  df$Pop <- x[2:9]
  df$Pop[which(df$Pop==0)] <- 1
  model1 <- lm(log(Pop) ~ Years, data = df)
  predicted <- exp(predict(model1, newdata = data.frame(Years = c(0:95))))
  return(predicted)
}

splinemodel <- function(x) {
  model1 <- smooth.spline(seq(0, 84, 12), x[2:9])
  return(predict(model1, 1:96)$y)
}

predictedPopDf <- data.frame(t(apply(TempPop, 1, splinemodel)))
predictedPopDf$ZipCodes <- TempPop$ZipCode

TempOhioZipCodeYear <- OpioidDeathsZipCodeYear
TempOhioZipCodeYear2 <- matrix(0,1,98)

for(i in as.numeric(predictedPopDf$ZipCodes)){
  if(!substring(as.character(i),1,1) == "*") {
    if(!i %in% TempOhioZipCodeYear$ZipCode){
      temp <- cbind(matrix(0,1,97), i)
      TempOhioZipCodeYear2 <- rbind(TempOhioZipCodeYear2, temp)
    } else {
      temp <- matrix(as.numeric(TempOhioZipCodeYear[TempOhioZipCodeYear$ZipCodes==i, ]), 1, 98)
      TempOhioZipCodeYear2 <- rbind(TempOhioZipCodeYear2, temp)
    }
```

```
  }
}

TempOhioZipCodeYear2 <- as.data.frame(TempOhioZipCodeYear2)
TempOhioZipCodeYear2 <- TempOhioZipCodeYear2[-1,]

names(TempOhioZipCodeYear2) <- names(OpioidDeathsZipCodeYear)
rownames(TempOhioZipCodeYear2) <- TempOhioZipCodeYear2$ZipCodes

POpDeaths <- merge(OpioidDeathsZipCodeYear[,-97], predictedPopDf, by = "ZipCodes")

aggOpioidsDeath <- data.frame(ZipCodes=POpDeaths$ZipCodes)
aggOpioidsPop <- data.frame(ZipCodes=POpDeaths$ZipCodes)

aggOpioidsDeath_ind <- data.frame(ZipCodes=POpDeaths$ZipCodes)
aggOpioidsPop_ind <- data.frame(ZipCodes=POpDeaths$ZipCodes)

aggOpioidsDeath_ind <- data.frame(ZipCodes=POpDeaths$ZipCodes)
aggOpioidsPop_ind <- data.frame(ZipCodes=POpDeaths$ZipCodes)

### AGGREGATION PREDIOD FOR TEMPORAL ANALYSIS 2 #####
aggregation_period <- 1
###-------------------------####

periodSize <- 96 / aggregation_period

for (subframe in seq(2, 98-aggregation_period, aggregation_period)) {
  ifelse(
   aggregation_period == 1,
   aggOOD <- POpDeaths[subframe],
   aggOOD <- apply(POpDeaths[,seq(subframe,subframe+aggregation_period-1)], 1, sum)
  )
  ifelse(
   aggregation_period == 1,
   avgPop <- POpDeaths[subframe+96],
   avgPop <- apply(POpDeaths[seq(subframe+96, subframe+96+aggregation_period-1)], 1, mean)
 )
 aggOpioidsDeath_ind[,colnames(POpDeaths)[subframe]] <- aggOOD
 aggOpioidsPop_ind[,colnames(POpDeaths)[subframe]] <- avgPop
}

CasesAdjusted_ind <- apply(aggOpioidsDeath_ind[,-1], 2, sum)
PopAdjusted_ind <- apply(aggOpioidsPop_ind[,-1], 2, sum)
ratesAdjusted_ind <- apply(aggOpioidsDeath_ind[,-1], 2, sum)/apply(aggOpioidsPop_ind[,-1], 2, su
m)*100000
timeseries_Real_T_ind <- ts(ratesAdjusted_ind, start = 1, end = length(CasesAdjusted_ind))

pvaluesig_ind <- c()
pvaluesig2_ind <- c()
pvaluecum_ind <- 1
pos_test_ind <- 0
modelbest_ind <- c()
modelbest_ind_report <- c()
```

```
period_ind <- 12
vector_cases <- as.data.frame(timeseries_Real_T_ind)
vector_cases$population <- PopAdjusted_ind

for (i in period_ind:(length(ratesAdjusted_ind)-period_ind)) {
   pre.period <- c(i-period_ind, i)
   post.period <- c(i+1, i+period_ind-1)
   impact <- CausalImpact(log(vector_cases), pre.period, post.period)
   pvalue <- impact$summary$p[1]
   pvaluesig_ind[length(pvaluesig_ind)+1] <- i
   pvaluesig2_ind[length(pvaluesig2_ind)+1] <- pvalue
   modelbest_ind[length(modelbest_ind)+1] <- impact
   modelbest_ind_report[length(modelbest_ind_report)+1] <- impact$report
}

trim_temp <- c()
for (i in 1:(96/3)) {
  for (j in 1:(12/4)){
    trim_temp[length(trim_temp)+1] <- i
  }
}

sem_temp <- c()
for (i in 1:(96/6)) {
  for (j in 1:(12/2)){
    sem_temp[length(sem_temp)+1] <- i
  }
}

year_temp <- c()
for (i in 1:(96/12)) {
  for (j in 1:(12/1)){
    year_temp[length(year_temp)+1] <- i
  }
}

temporaltrend <- data.frame(monthpvalue = pvaluesig2_ind)
agg_period <- trim_temp
temporaltrend$agg <- agg_period[seq(period_ind,length(ratesAdjusted_ind)-period_ind,1)]
plot_effect <- aggregate(monthpvalue ~ agg, temporaltrend, mean)

### AGGREGATION PREDIOD #####
aggregation_period <- 3
###-------------------------####

periodSize <- 96 / aggregation_period

for (subframe in seq(2, 98-aggregation_period, aggregation_period)) {
  ifelse(
    aggregation_period == 1,
    aggOOD <- POpDeaths[subframe],
    aggOOD <- apply(POpDeaths[,seq(subframe,subframe+aggregation_period-1)], 1, sum)
  )
  ifelse(
```

```
    aggregation_period == 1,
    avgPop <- POpDeaths[subframe+96],
    avgPop <- apply(POpDeaths[seq(subframe+96, subframe+96+aggregation_period-1)], 1, mean)

  )
  aggOpioidsDeath[,colnames(POpDeaths)[subframe]] <- aggOOD
  aggOpioidsPop[,colnames(POpDeaths)[subframe]] <- avgPop
}

MapCasesTime <- aggOpioidsDeath
MapPopTime <- aggOpioidsPop

CasesAdjusted <- apply(aggOpioidsDeath[,-1], 2, sum)
PopAdjusted <- apply(aggOpioidsPop[,-1], 2, sum)
ratesAdjusted <- apply(aggOpioidsDeath[,-1], 2, sum)/apply(aggOpioidsPop[,-1], 2, sum)*100000

timeseries_Real_T <- ts(CasesAdjusted, start = 1, end = length(CasesAdjusted))
startint <- round(length(CasesAdjusted)*0.3)
endint <- round(length(CasesAdjusted)*0.8)

pvaluesig <- c()
pvaluesig2 <- c()
pvaluecum <- 1
pos_test <- 0

for (i in startint:endint) {
  curvetoTest <-  timeseries_Real_T ### Cases Included
  timeseries_Real_T2 <- curvetoTest
  balance <- i
  dummy_TS <- c(rep(0,balance), rep(1,periodSize-balance))
  test_interrupted <- as.data.frame(cbind(timeseries_Real_T2 + 0.0001, dummy_TS, log(PopAdjuste
d)))
  names(test_interrupted) <- c("OODeaths", "Test", "LogPopulation")
  test_interrupted$time <- 1:periodSize
  model2 <- glm(data = test_interrupted, OODeaths ~ Test*time + time + LogPopulation, family = p
oisson)
  pvalue <-summary(model2)$coefficients[,4][2]
  pvaluesig[length(pvaluesig)+1] <- i
  pvaluesig2[length(pvaluesig2)+1] <- pvalue
  if(pvalue<pvaluecum){
    modelbest <- model2
    pos_test <- balance
    pvaluecum <- pvalue
    summaryTest <- summary(model2)
  }
}

par(mfrow=c(1,1))
plot (
  curvetoTest / PopAdjusted * 10^5,
  type = 'p',
  xaxt = "n",
  yaxt = "n",
  xlab = "",
```

```
  ylab = "",
  cex.lab = 2,
  ylim = c(-3, max(curvetoTest / PopAdjusted * 10^5))
)

plot_effects_sig <- plot_effect[plot_effect$monthpvalue<0.005,]
temp <- rbind(plot_effects_sig[1,],
        plot_effects_sig[c(FALSE,!diff(plot_effects_sig$agg)==1),]
)

for (sig_time in temp$agg){
  print(sig_time)
  rect(sig_time-1, -10, sig_time+1, max(curvetoTest)+10, col = "#55555522",border = "transparen
t")
}

k <- 0
lastk <- 0
for (element in agg_period[seq(period_ind,length(ratesAdjusted_ind)-period_ind,1)]){
  if(element %in% temp$agg){
    if(!k-lastk == 1){
      tempTrend <- data.frame(effect = modelbest_ind[[k]]$cum.effect)
      tempTrend$agg <- agg_period
      plot_cum_effect <- aggregate(effect ~ agg, tempTrend, sum, na.action=NULL)
      lines(
        plot_cum_effect$agg,
        plot_cum_effect$effect,
          type = "o",
          col="black",
          pch=18,
          lwd=2,
          lty=1,
          cex=1
      )
      print(modelbest_ind_report[k])
    }
    lastk <- k
  }
  k <- k+1
}

datanew <- data.frame(LogPopulation=log(PopAdjusted), time = 1:periodSize, Test=0)
datanew_2 <- data.frame(LogPopulation=log(PopAdjusted), time = 1:periodSize, Test=1)

pred1 <- predict(modelbest, type="response", newdata = datanew) / PopAdjusted * 10^5
pred2 <- predict(modelbest, type="response", newdata = datanew_2) / PopAdjusted * 10^5
pred1 <- ifelse(pred1>0, pred1, 0.00001)
pred2 <- ifelse(pred2>0, pred2, 0.00001)

lines(
  1:periodSize,
  pred1[1:periodSize],
  type = "o",
  col="#0C92C2FF",
```

```
  pch=18,
  lwd=3,
  lty=1,
  cex=1
)

y <- plot_effect[,2]
n <- length(y)
x <- plot_effect[,1]
s = smooth.spline(x, y, spar=0.1)
xy <- predict(s, seq(min(x), max(x), by=1))
m <- length(xy$x)
x.poly <- c(xy$x, xy$x[m], xy$x[1])
y.poly <- c(xy$y+0.01, 0, 0)
polygon(
  x.poly,
  y.poly * max(curvetoTest / PopAdjusted * 10^5),
  col="#0C92C222",
  border=NA
)

axis(1, at=seq(1,periodSize), labels=as.character(colnames(POpDeaths)[seq(2, 98-aggregation_peri
od, aggregation_period)]), las=2, cex.axis=1)
axis(2, cex.axis=1)
stepsize <- seq(0, max(curvetoTest / PopAdjusted * 10^5), 5)
axis(4, at=stepsize, labels=round(seq(0, max(stepsize)/max(curvetoTest / PopAdjusted * 10^5), le
ngth.out=length(stepsize)), 2),  cex.axis=1)

legend("topleft",
       legend=c("Rates Prescription opioid overdose deaths",
                "Trendline (Poisson)",
                "Turning points estimated effect",
                "p-values simulated turning points (right axis)",
                "Significant turning points periods (p-value < 0.005)"
                ),
       col=c("black", "#0C92C2FF", "black", "#0C92C222", "#55555522"),
       lty=c(NA, 2, 2, NA, NA),
       lwd=c(NA, 2, 1, NA, NA),
       cex=1,
       pch=c(1, 18, 18, 17, 15),
       bty = "n",
       pt.cex = c(1,1,1,2,2)
)
```

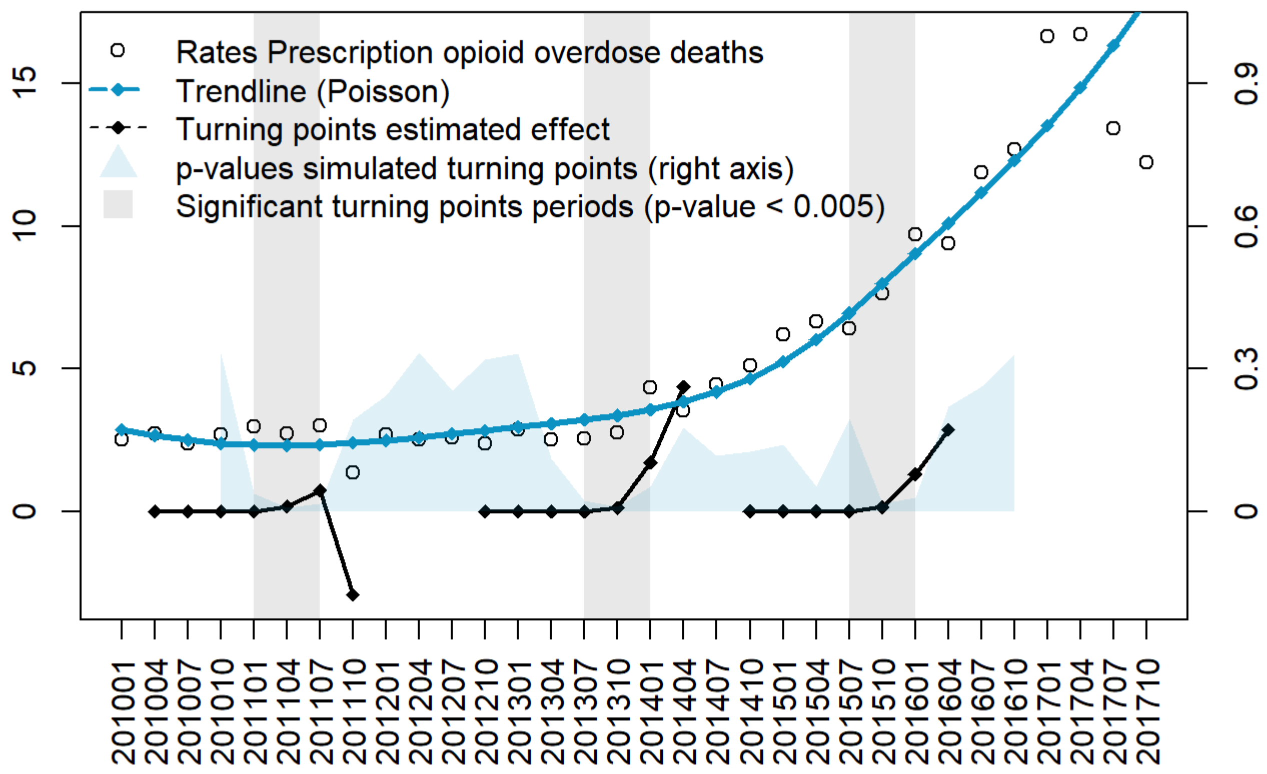

Rates Prescription opioid overdose deaths
Trendline (Poisson)
Turning points estimated effect
p-values simulated turning points (right axis)
Significant turning points periods (p-value < 0.005)
0
5
10
15
0
0.3
0.6
0.9
201001
201004
201007
201010
201101
201104
201107
201110
201201
201204
201207
201210
201301
201304
201307
201310
201401
201404
201407
201410
201501
201504
201507
201510
201601
201604
201607
201610
201701
201704
201707
201710
